# Supplementary material for: The Proinflammatory Cytokine IL-36γ Is a Global Discriminator of Harmless Microbes and Invasive Pathogens within Epithelial Tissues
Source: Cell Rep. 2020 Dec 15;33(11):108515. doi: 10.1016/j.celrep.2020.108515 (PMC7758160; doi:10.1016/j.celrep.2020.108515)
Supplement: Document S1. Figures S1–S5 [file mmc1.pdf]

Cell Reports, Volume *33*

Supplemental Information

The Proinflammatory Cytokine IL-36γ Is a Global Discriminator of Harmless Microbes and Invasive Pathogens within Epithelial Tissues

Thomas Macleod, Joseph S. Ainscough, Christina Hesse, Sebastian Konzok, Armin Braun, Anna-Lena Buhl, Joerg Wenzel, Paul Bowyer, Yutaka Terao, Sarah Herrick, Miriam Wittmann, and Martin Stacey

Supplementary Information

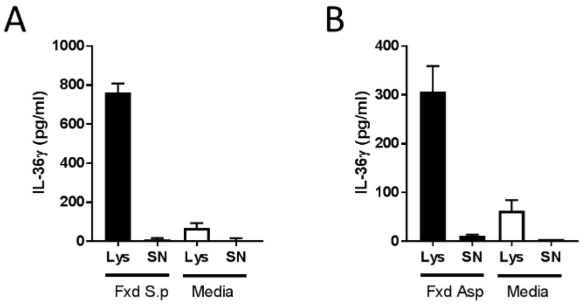

Fig. S1*: TR146 cells do not release IL-36γ following non-viable pathogen stimulation,* related to Figure 1. TR146 cells (10^5 per well) were treated with fixed *S. pyogenes* (**A**; Fxd S.p), fixed *A. fumigatus* conidia **(B**; Fxd Asp) or media alone for 24 hours. Lysate and supernatant IL-36γ concentration measured by ELISA. Data shown are mean ± SEM (n = 3).

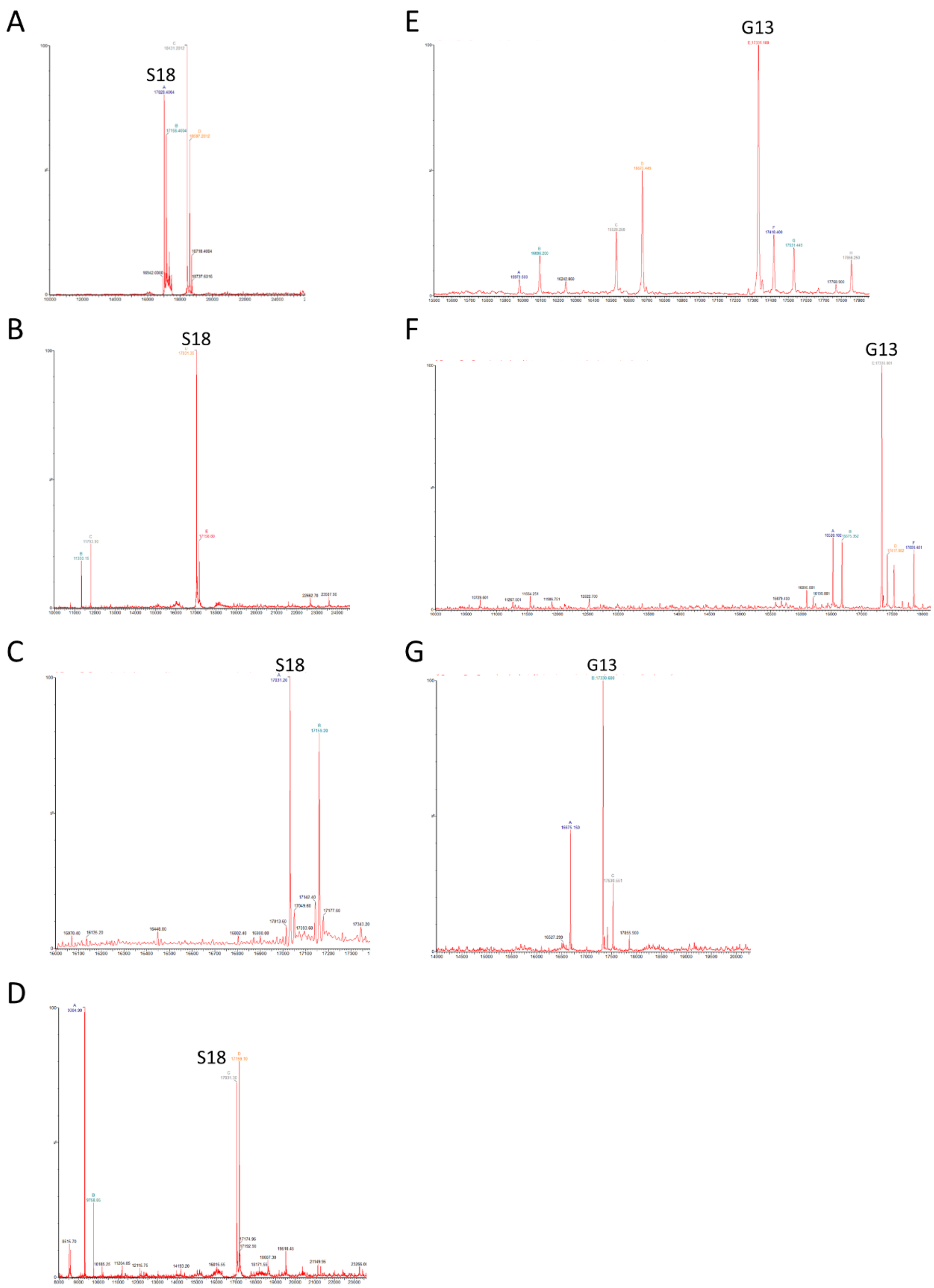

Fig. S2: *Liquid chromatography-mass spectrometry traces of IL-36 cleavage*, related to Figure 2. 2 µg of recombinant full length IL-36 proteins were incubated with culture filtrates or recombinant proteases for 1 hour at 37C and analysed by liquid chromatography-mass spectrometry as outlined in methods and materials. Liquid chromatography traces show resulting cleavage products from hIL-36γ + *A. fumigatus* (**A**), hIL-36γ + *T. rubrum* (**B**), hIL-36γ + *S. pyogenes* (**C**)**,** hIL-36γ + *S. areus* (**D**)**,** mIL-36γ + *A. fumigatus* (**E**), mIL-36γ + *T. rubrum* (**F**), mIL-36γ + *S. pyogenes* (**G**). Peaks corresponding to the active IL-36 truncations are depicted above and labelled with their respective N-terminal amino acids. hIL-36γ S18 (S18; 17031 Da), mIL-36γ G13 (G13; 17331 Da).

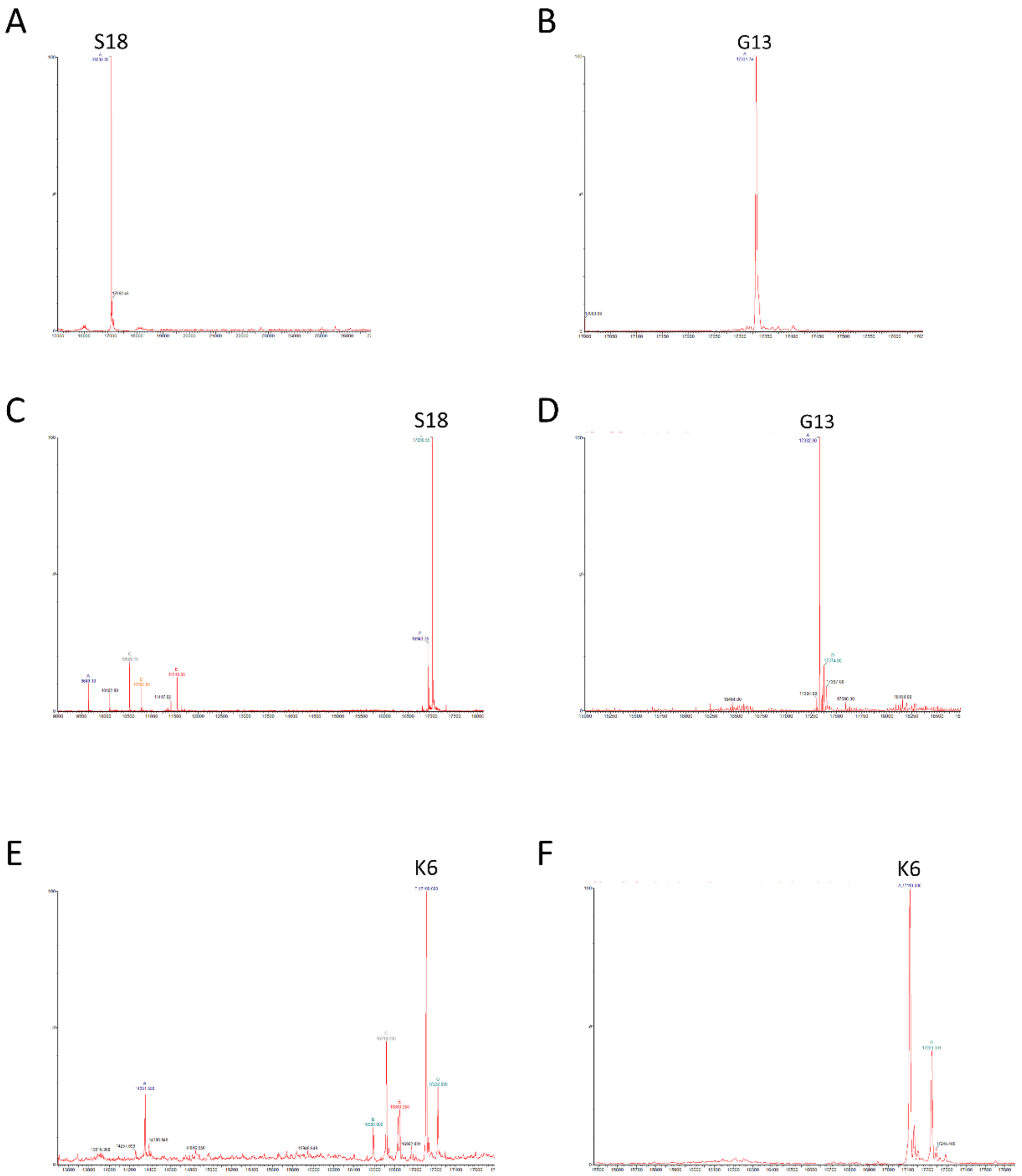

Fig. S3: *Liquid chromatography-mass spectrometry traces of IL-36 cleavage*, related to Figure 2. 2 µg of recombinant full length IL-36 proteins were incubated with culture filtrates or recombinant proteases for 1 hour at 37C and analysed by liquid chromatography-mass spectrometry as outlined in methods and materials. Liquid chromatography traces show resulting cleavage products from hIL-36γ + SpeB (**A**), mIL-36γ + SpeB (**B**), hIL-36γ + Asp F13 (**C**), mIL-36γ + Asp F13 (**D**), hIL-36α + *A. fumigatus* (**E**), hIL-36α + *T. rubrum* (**F**). Peaks corresponding to the active IL-36 truncations are depicted above and labelled with their respective N-terminal amino acids. hIL-36γ S18 (S18; 17031 Da), mIL-36γ G13 (G13; 17331 Da), hIL-36α K6 (K6; 17113).

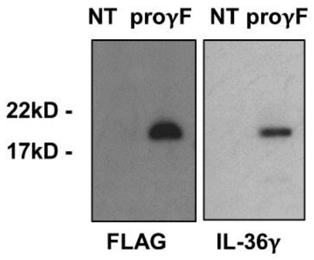

Fig. S4: *Confirmation of FLAG-tagged IL-36γ expression by stable proγF-293 cell line,* related to STAR Methods. Lysates of non-transfected 293 cells (NT) and stable proγF-293 cells (proγF) were analysed for expression of FLAG-tagged IL-36γ by western blot.

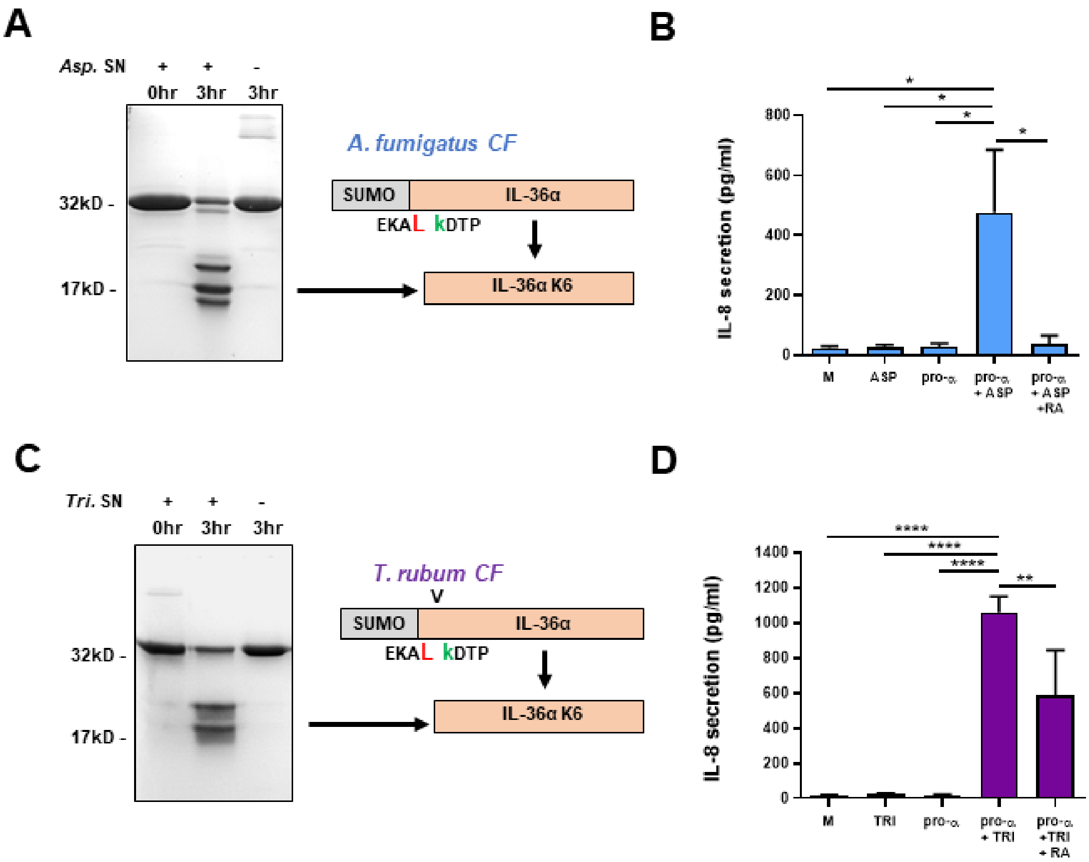

***Fig. S5**: IL-36a is also cleaved and activated by Aspergillus fumigatus and Trichophyton rubrum*, related to Figure 2. 1 µg of SUMO-tagged human IL-36α was incubated at 37˚C for 0 hours or 3 hours, with or without 2 µl of either *A. fumigatus* culture filtrate (Asp. CF; **A**) or *T. rubrum* culture filtrate (*Tri.* CF; **C**). Samples were analysed by Coomassie stained SDS-PAGE gel. Cleaved products were also analysed by mass spectrometry, with diagrams depicting the IL-36α truncation generated in response to each culture filtrate. In addition, HaCaT cells (10^5 per well) were incubated for 24 hours with media alone (M), pro-IL-36α (pro-α; 10 nM), culture filtrate, a combination of pro-IL-36α and culture filtrate, or a combination of pro-IL-36α, culture filtrate and IL-36RA (50 nM; **B, D**). A one-way ANOVA was used to determine statistical significance of differences between treatment groups. *$p < 0.05$, **$p<0.01$, ****$p < 0.0001$. Data shown are mean ± SEM (**B**; n=3, **D**; n=4).
