# Supplementary material for: The Proinflammatory Cytokine IL-36γ Is a Global Discriminator of Harmless Microbes and Invasive Pathogens within Epithelial Tissues
Source: Cell Rep. 2020 Dec 15;33(11):108515. doi: 10.1016/j.celrep.2020.108515 (PMC7758160; doi:10.1016/j.celrep.2020.108515)
Supplement: Document S2. Article plus Supplemental Information [file mmc2.pdf]

Cell Reports

Article

The Proinflammatory Cytokine IL-36γ Is a Global Discriminator of Harmless Microbes and Invasive Pathogens within Epithelial Tissues

Graphical Abstract

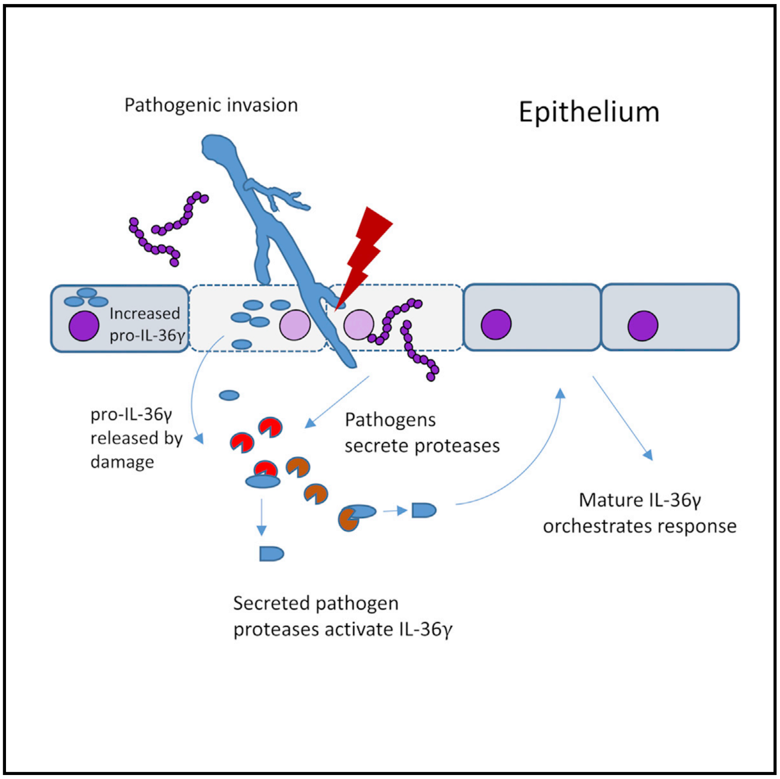

Highlights

- Epithelial pathogens induce expression and release of IL-36γ
- Proteases secreted by several epithelial pathogens activate IL-36γ
- The *A. fumigatus* and *S. pyogenes* virulence factors Asp F13 and SpeB activate IL-36γ
- IL-36γ is a global sensor of pathogen-derived proteases during epithelial infection

Authors

Thomas Macleod, Joseph S. Ainscough, Christina Hesse, ..., Sarah Herrick, Miriam Wittmann, Martin Stacey

Correspondence

m.wittmann@leeds.ac.uk (M.W.), m.stacey@leeds.ac.uk (M.S.)

In Brief

Macleod et al. demonstrate that the epithelial cytokine IL-36γ discriminates between pathogens and harmless commensals at epithelial barriers. IL-36γ is upregulated by epithelial tissue following microbial challenge, but it is only released and activated by destructive pathogenic microbes, whereupon it initiates an immune response.

Macleod et al., 2020, Cell Reports *33*, 108515
December 15, 2020 Crown Copyright © 2020
https://doi.org/10.1016/j.celrep.2020.108515

CellPress

Cell Reports

CellPress
OPEN ACCESS

Article

The Proinflammatory Cytokine IL-36γ Is a Global Discriminator of Harmless Microbes and Invasive Pathogens within Epithelial Tissues

Thomas Macleod,[1] Joseph S. Ainscough,[1] Christina Hesse,[2,3] Sebastian Konzok,[2,3] Armin Braun,[2,3] Anna-Lena Buhl,[4] Joerg Wenzel,[4] Paul Bowyer,[5] Yutaka Terao,[6] Sarah Herrick,[7] Miriam Wittmann,[1,8,9,*] and Martin Stacey[1,10,*]

[1]Faculty of Biological Sciences, School of Molecular and Cellular Biology, University of Leeds, Leeds LS2 9JT, UK
[2]Fraunhofer Institute for Toxicology and Experimental Medicine, Nikolai-Fuchs-Straße 1, 30625 Hannover, Germany
[3]Member of the German Center for Lung Research (DZL), Biomedical Research in Endstage and Obstructive Lung Disease Hannover (BREATH) Research Network, Hannover, Germany
[4]Department of Dermatology and Allergy, University of Bonn, Bonn 53012, Germany
[5]Division of Infection, Immunity & Respiratory Medicine, University of Manchester, Manchester M13 9PL, UK
[6]Division of Microbiology and Infectious Diseases, Niigata University Graduate School of Medical and Dental Sciences, Niigata, Japan
[7]Faculty of Biology Medicine and Health, School of Biological Sciences, University of Manchester and Manchester Academic Health Science Centre, Manchester M13 9PL, UK
[8]Faculty of Medicine and Health, Leeds Institute of Rheumatic and Musculoskeletal Medicine, University of Leeds, Leeds LS2 9JT, UK
[9]Leeds Biomedical Research Centre, National Institute for Health Research, Leeds Teaching Hospitals, Leeds, UK
[10]Lead Contact
*Correspondence: m.wittmann@leeds.ac.uk (M.W.), m.stacey@leeds.ac.uk (M.S.)
https://doi.org/10.1016/j.celrep.2020.108515

SUMMARY

Epithelial tissues represent vital interfaces between organisms and their environment. As they are constantly exposed to harmful pathogens, innocuous commensals, and environmental microbes, it is essential they sense and elicit appropriate responses toward these different types of microbes. Here, we demonstrate that the epithelial cytokine interleukin-36γ (IL-36γ) acts as a global discriminator of pathogenic and harmless microbes via cell damage and proteolytic activation. We show that intracellular pro-IL-36γ is upregulated by both fungal and bacterial epithelial microbes; yet, it is only liberated from cells, and subsequently processed to its mature, potent, proinflammatory form, by pathogen-mediated cell damage and pathogen-derived proteases. This work demonstrates that IL-36γ senses pathogen-induced cell damage and proteolytic activity and is a key initiator of immune responses and pathological inflammation within epithelial tissues. As an apically located epithelial proinflammatory cytokine, we therefore propose that IL-36γ is critical as the initial discriminator of harmless microbes and invasive pathogens within epithelial tissues.

INTRODUCTION

As crucial interfaces between the body and its environment, epithelial sites such as the skin, gut, and lungs are constantly challenged by ubiquitous exogenous microbes. One mechanism by which the host can potentially discriminate harmful pathogens from commensals is via pathogenic damage. As host tissue can be a non-permissive environment for microbial growth, pathogens often produce cytotoxic virulence factors that assist colonization by facilitating immunosuppression and nutrient acquisition. Damaging virulence factors result in the release of cellular components known as alarmins such as interleukin-1α (IL-1α) and high mobility group box 1 (HMGB1), which are normally sequestered within healthy cells. Upon release, these function to activate tissue-resident inflammatory cells and induce cytokine production that recruits inflammatory cells to the site of infection and skews the adaptive immune system toward an appropriate response to ultimately clear infection (Bianchi et al., 2017; Kono et al., 2010; Eigenbrod et al., 2008; Oppenheim and Yang, 2005; Yang et al., 2017).

Interestingly, a significant number of epithelial pathogens have evolved proteolytic enzymes for nutrient acquisition and the colonization of extracellular matrix (ECM)-rich epithelial tissues (Singh et al., 2012). For example, the clinically significant bacterial pathogen *Streptococcus pyogenes*, an etiological agent of pharyngitis, cellulitis, and erysipelas, and *Aspergillus fumigatus*, which causes invasive aspergillosis with fatality rates in excess of 50% in immunocompromised individuals (Lin et al., 2001), both produce ECM-degrading proteases (Tamura et al., 2004; Burns et al., 1996; Iadarola et al., 1998). Given that these proteases are important mediators of invasion and that the ability for a microbe to invade can be the difference between a pathogen and a commensal, it is postulated that proteases are often essential mediators of pathogenicity. It would therefore be beneficial to the host to identify the presence of such proteolytic virulence factors, enabling immediate recognition of a pathogenic presence.

This is an open access article under the CC BY license (http://creativecommons.org/licenses/by/4.0/).

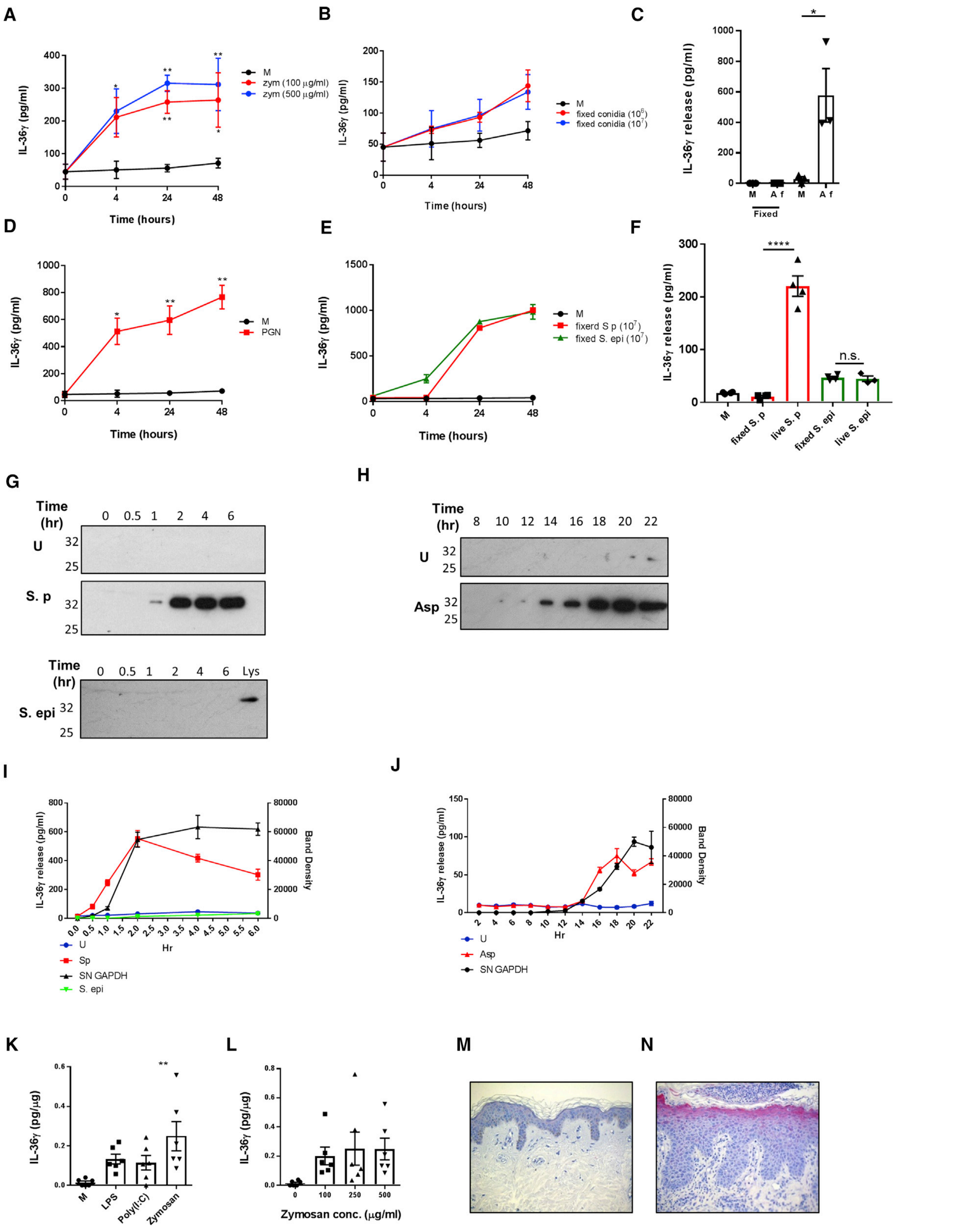

(legend on next page)

The IL-36 cytokines (IL-36α, IL-36β, IL-36γ, receptor antagonist [RA]) are a recently characterized group of cytokines belonging to the IL-1 superfamily (Dunn et al., 2001). While many IL-1 cytokines can be found expressed throughout the body, the IL-36 cytokines are predominately expressed in epithelial tissue such as the skin, lungs, gut, and mucosa, particularly at apical locations, suggesting an important role in barrier immune function (Dunn et al., 2001; Gabay and Towne, 2015; Boutet et al., 2016). Indeed, it is now well established that IL-36γ is a proinflammatory mediator highly expressed in psoriasis and is involved in the initiation and maintenance of pathological inflammation (Johnston et al., 2011; He et al., 2013; Berekmeri et al., 2018; Bridgewood et al., 2018). However, this cytokine is also a critical mediator of immune responses to several classes of invading pathogens at epithelial barriers (Kovach et al., 2017; Gao et al., 2018). As a proinflammatory protein, IL-36γ induces expression of a range of antimicrobial peptides such as cathelicidin (LL-37), human beta defensins 2 and 3, and S100 proteins as well as several cytokines and chemokines such as itself and IL-1α/β, IL-8, IL-17, and CCL20, cytokines typically associated with an immune response to extracellular pathogens (Foster et al., 2014; Johnston et al., 2011; Carrier et al., 2011). Intratracheal administration of IL-36γ in mice leads to an influx of neutrophils and macrophages, in addition to T helper 1 (Th1) and Th17 cells, crucial for the orchestration of an adaptive immune response to invading pathogens (Ramadas et al., 2011; Carrier et al., 2011). Furthermore, several studies have shown expression of IL-36γ is elevated upon challenge by numerous invasive bacterial and fungal pathogens, including *Streptococcus pneumoniae*, *Klebsiella pneumoniae*, *Aspergillus fumigatus*, and *Candida albicans*, and subsequent studies have implicated IL-36γ involvement in initiating an inflammatory response following infection by such organisms. Indeed, in mouse models of bacterial pneumonia and mucosal candidiasis, deficiency of IL-36 signaling leads to increased mortality and fungal burdens (Kovach et al., 2017; Gresnigt et al., 2013; Verma et al., 2018; Braegelmann et al., 2018).

As with other IL-1 family cytokines, IL-36γ is produced as an inactive precursor that requires precise N-terminal processing to become biologically active. The activating cleavage event occurs between Gln_{17} and Ser_{18}, nine amino acids upstream of the conserved IL-1 motif, and deviation from this site by a single amino acid reduces biological activity more than 1,000-fold (Towne et al., 2011). Unlike the well-characterized IL-1β and IL-18 cytokines, this activation has been shown to be an inflammasome-independent process, which, in psoriasis, is mediated via the endogenous proteases cathepsin S and neutrophil elastase (Ainscough et al., 2017; Henry et al., 2016). However, the mechanisms by which IL-36γ is released and activated during microbial infection remain unknown.

Given the importance of proteolytic virulence factors in establishing pathogenic invasion, and the importance of proteolytic processing in the activation of IL-36γ at epithelial barriers, we examined the effects of pathogen-mediated release and proteolytic activation of IL-36γ. The work presented here provides evidence that IL-36γ acts as a link between pathogenic proteolytic activity and initiation of an immune response in epithelial tissue. We demonstrate that IL-36γ is upregulated by microbes, released by pathogenic damage, and processed into its bioactive form by several proteases from important fungal and bacterial epithelial pathogens. We therefore propose that IL-36γ is an epithelial alarmin that acts as a global early sensor of pathogenic invasion, enabling the innate immune response to discriminate harmful from harmless microbes. This work builds a more complete picture of how IL-36 signaling is initiated during microbial infection and demonstrates the importance of IL-36 cytokines in immune defense at epithelial barriers.

RESULTS

Epithelial Pathogens Upregulate and Release IL-36γ

IL-36γ is expressed at epithelial barriers and has been shown to be upregulated in response to a number of pathogen-associated molecular patterns (PAMPs), such as lipopolysaccharide (LPS), β-glucans, and poly(I:C). While mechanisms of release of IL-36γ are currently unclear, one possibility is that IL-36γ remains in the cytoplasm until cellular damage results in release of the immature protein into the surrounding extracellular space, where it is available for activation by extracellular proteases.

To investigate the effect pathogenic challenge has on IL-36γ expression and release, the oral buccal epithelial cell line TR146 was stimulated with varying concentrations of zymosan and peptidoglycan for 0–48 h (Figures 1A and 1D). Measurement of lysate IL-36γ by ELISA showed both zymosan- and peptidoglycan-induced expression of IL-36γ. TR146 cells were also treated with a varying amount of fixed *A. fumigatus* conidia, *S. pyogenes*, and the harmless commensal *Staphylococcus epidermidis* for 0–48 h, and lysate IL-36γ was measured by ELISA (Figures 1B and 1E). Again, an increase in IL-36γ expression was observed upon stimulation with all fixed microbes.

Figure 1. Epithelial Pathogens Induce Expression and Release of IL-36γ

(A–N) TR146 cells (10^5 per well) were treated with M, zym (A; 100 and 500 μg/mL), or fixed *Aspergillus* conidia (B; 10^6 and 10^7) for 0–48 h, and lysate IL-36γ was measured by ELISA (n = 3). (C) TR146 cells were treated with M or with either live or fixed *Aspergillus* conidia (10^7) for 24 h, and supernatant IL-36γ was measured by ELISA (n = 3). TR146 cells were treated with PGN (D; 10 μg/mL), fixed S. p, or fixed S. epi (E) (10^7) for 0–48 h, and lysate IL-36γ was measured by ELISA (n = 3). TR146s (F) were treated for 24 h with M or with either live or fixed S. p and S. epi, and supernatant IL-36γ was measured by ELISA (n = 4). TR146 cells were subjected to media change with antibiotic-free DMEM and then left U, inoculated with either S. p (10^7) or S. epi (10^7) (supernatant GAPDH compared with Lys) for 0–6 h (G, S. p; I, S. epi) or Asp (10^6) for 0–22 h (H and J). Supernatant was collected from cells at the indicated time points and measured for GAPDH by western blot (G and H) and IL-36γ by ELISA (I and J) (n = 3). GAPDH band intensity was analyzed in ImageJ software and plotted against IL-36γ concentration (I and J). Precision-cut lung slices were treated with PAMPs (K) and zym (L; 100–500 μg/mL). Lysate IL-36γ was measured by ELISA (n = 6). Anti-IL-36γ immunohistochemistry staining of sections from healthy (M) and *T. rubrum*-infected (N) skin. Western blots are representative of three individual experiments. A one-way ANOVA was used to determine statistical significance of differences between treatment groups. *$p < 0.05$, **$p < 0.01$, and ****$p < 0.0001$. Data shown are mean ± SEM. Abbreviations are as follows: Asp, *A. fumigatus* conidia; Lys, lysate GAPDH; PGN, peptidoglycan; M, media alone; S. epi, *S. epidermidis*; S. p, *S. pyogenes*; U, uninfected; zym, zymosan.

However, while IL-36γ expression was evident in the lysate, little or no corresponding increase was observed in the supernatant of stimulated cells, with IL-36γ often undetectable (Figure S1). TR146 cells were then treated with either fixed or viable *A. fumigatus* conidia, *S. pyogenes*, or *S. epidermidis* (Figures 1C and 1F). Measurement of IL-36γ in supernatants from cells treated with live *A. fumigatus* and *S. pyogenes*, both of which are pathogens, showed a significant increase in extracellular IL-36γ compared with their fixed counterparts, indicating release of the cytokine only occurred when cells were inoculated with viable pathogens. Strikingly, cells treated with the commensal *S. epidermidis* did not release IL-36γ into their culture supernatant, suggesting the live commensal was unable to induce IL-36γ release.

Given that extracellular IL-36γ was only observed following inoculation with live proliferating pathogens and that bacterial and fungal growth can be destructive to cells, it was hypothesized that cellular damage was responsible for release of IL-36γ. To test this, TR146 cells were infected with either *A. fumigatus*, *S. pyogenes*, or *S. epidermidis*, and the release of the cytosolic housekeeping protein glyceraldehyde 3-phosphate dehydrogenase (GAPDH) was monitored alongside that of IL-36γ. As shown in Figures 1G and 1H, extracellular GAPDH was present only in the pathogen-infected samples, and release of both GAPDH and IL-36γ coincided over time (Figures 1I and 1J), suggesting release of IL-36γ may occur as a result of membrane damage. We did not observe the release of either IL-36γ or GAPDH from cells infected with the commensal *S. epidermidis*, suggesting this commensal is not inducing release of IL-36 as it is not inducing cellular membrane damage.

To examine the relevance of IL-36γ in other types of epithelial tissues, the expression of IL-36γ in response to PAMP stimulation was examined in cultured human lung tissue using *ex vivo* precision-cut lung slices (Temann et al., 2017). Slices were treated with LPS, poly(I:C), and zymosan at the indicated concentrations, and lysate IL-36γ was assessed by IL-36γ ELISA. As shown in Figure 1G, all PAMPs tested induced increased expression of IL-36γ, with zymosan inducing the strongest expression (Figures 1K and 1L). Again, no measurable IL-36γ was detectable in the supernatants of the precision-cut lung slices. Furthermore, immunohistochemistry on sections from healthy and *Trichophyton rubrum*-infected skin shows IL-36γ is highly expressed in the uppermost layers of the epidermis following infection by *T. rubrum* (Figures 1M and 1N).

IL-36γ Is Activated by Proteases Derived from a Range of Human Pathogens

As many epithelial pathogens are known to express proteases to facilitate invasion and nutrient acquisition, the effects of culture filtrates from *A. fumigatus*, *S. pyogenes*, *T. rubrum*, and *Staphylococcus aureus* on the processing of IL-36γ were investigated.

Recombinant pro-IL-36γ possessing an N-terminal small ubiquitin-like modifier (SUMO) tag was incubated with culture filtrates from *A. fumigatus*, *S. pyogenes*, *T. rubrum*, and *S. aureus* at 37°C for 1 h. Resolution by SDS-PAGE revealed that incubation with the culture filtrates from all pathogens tested results in truncation of IL-36γ (Figures 2A–2D). Analysis of the truncated products by liquid chromatography-mass spectrometry (LC-MS) identified the prominent species as possessing a mass of 17,031 Da in all of the culture filtrates (Figure S2). This species corresponds to the predicted mass of the highly active mature IL-36γ S18 (illustrated in Figure 2). In order to confirm that N-terminal SUMO was not having an effect on cleavage, the culture filtrate incubations were repeated using recombinant pro-IL-36γ without N-terminal SUMO and analyzed by LC-MS. Again, the prominent species possessed a mass corresponding to mature IL-36γ S18 in all culture filtrates (Figure S2).

Culture filtrate-incubated pro-IL-36γ was tested for biological activity by utilizing an activity assay previously developed (Ainscough et al., 2017). Culture filtrates incubated with or without pro-IL-36γ were added to IL-36γ-sensitive HaCaT cells in the presence or absence of IL-36RA and monitored for IL-8 expression. While addition of either culture filtrate or pro-IL-36γ alone elicited only very low amounts of IL-8 secretion, the addition of culture filtrate and pro-IL-36γ induced strong responses (Figures 2E–2H). Furthermore, this response is shown to be IL-36 specific as addition of IL-36RA significantly reduced IL-8 secretion. These observations indicate several pathogens across different domains of life secrete proteases that activate IL-36γ.

In addition to human (h)IL-36γ, we also examined the susceptibility of mouse (m)IL-36γ to activation by these epithelial pathogens to test whether this activation is evolutionarily conserved. While hIL-36γ and mIL-36γ are structurally similar, the primary amino acid sequences surrounding the cleavage site required for generating biologically active IL-36γ are distinct.

As with hIL-36γ, mIL-36γ was incubated with culture filtrate of *A. fumigatus*, *S. pyogenes*, and *T. rubrum* at 37°C for 1 h prior to resolution by SDS-PAGE. As with hIL-36γ, cleavage was observed in all samples (Figures 3A–3C). While addition of pro-mIL-36γ or culture filtrate alone to mIL-36-sensitive mouse embryonic fibroblasts (MEFs) did not induce significant secretion of mIL-6, addition of culture-filtrate-incubated pro-mIL-36γ induced strong mIL-6 secretion (Figures 3D–3F). LC-MS analysis again confirmed that IL-36γ was cleaved to the proactive form of murine IL-36γ (G13).

Activation of IL-36γ by *S. pyogenes* Is Dependent upon Virulence Factor SpeB

Upon identifying culture filtrates extracted from several important human epithelial pathogens activated IL-36γ, we next endeavored to identify what was responsible for the activation. We proceeded with *S. pyogenes* and *A. fumigatus* as these pathogens are the most destructive and medically important of those originally tested.

In order to identify the *S. pyogenes* protease responsible for cleavage and activation of IL-36γ, SUMO-pro-IL-36γ was incubated with *S. pyogenes* culture filtrate in the presence of the broad-range Ser inhibitor PMSF, Cys inhibitor E64, and metalloproteinase inhibitor EDTA for 1 h at 37°C. As shown in Figure 4A, while cleavage was unaffected by addition of PMSF and EDTA, addition of the Cys protease inhibitor E64 completely inhibited cleavage. These samples were also tested for biological activity. Initially, protease inhibitors were added to cells with IL-36γ S18 to ensure they did not generate false negative results. A pan-protease inhibitor cocktail including E64, PMSF, and EDTA (PI) added to HaCaT cells in combination with IL-36γ S18 had no

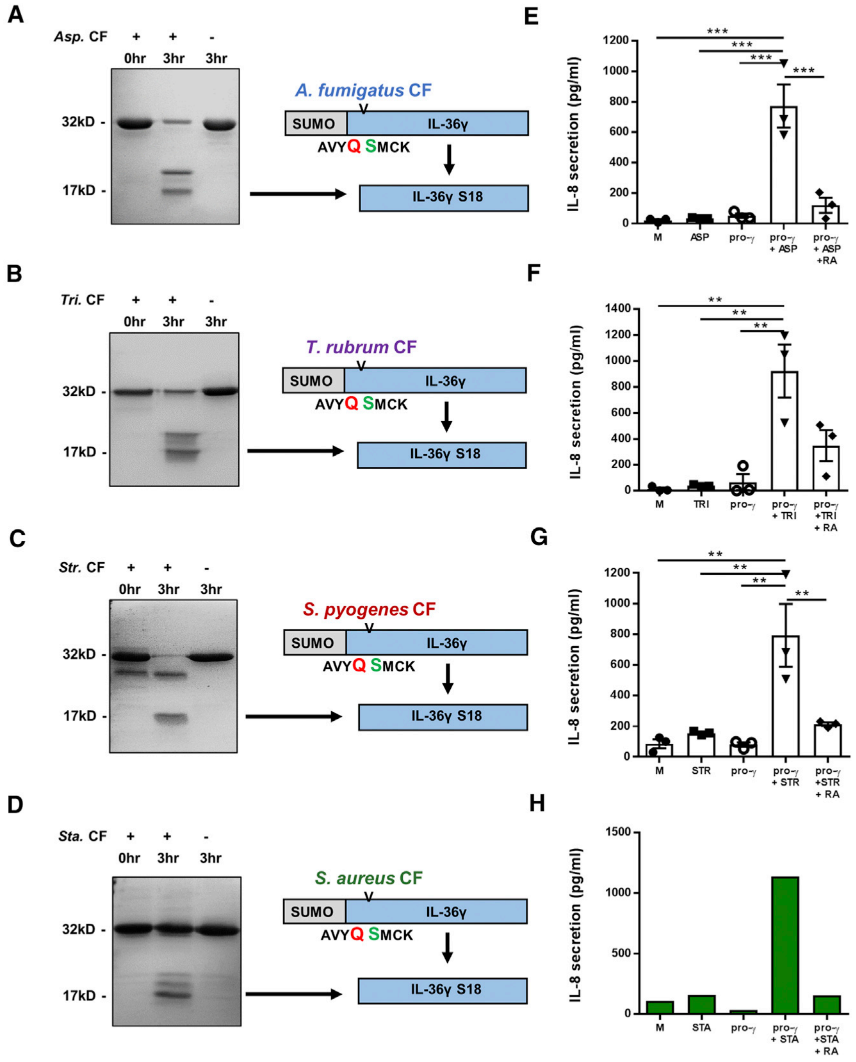

Figure 2. Human IL-36γ Is Activated by Proteases Derived from a Range of Human Pathogens

(A–H) SUMO-tagged human IL-36γ (1 μg) was incubated at 37°C for 0 or 3 h, with or without 2 μL of either *Asp*. CF (A), *Tri.* CF (B), *Str*. CF (C), or *Sta*. CF (D). Samples were analyzed by Coomassie-blue-stained SDS-PAGE gel. Cleaved products were also analyzed by mass spectrometry, with diagrams depicting the IL-36γ truncation generated in response to each CF. In addition, HaCaT cells (10^5 per well) were incubated for 24 h with M; pro-γ (10 nM); CF; a combination of pro-γ and CF; or a combination of pro-γ, CF, and IL-36RA (50 nM; E–H) (E–G, n = 3; H, n = 1). A one-way ANOVA was used to determine statistical significance of differences between treatment groups. **p < 0.01 and **p < 0.001. Data shown are mean ± SEM. Abbreviations are as follows: *Asp*. CF, *A. fumigatus* culture filtrate; pro-γ, pro-IL-36γ; *Sta*. CF, *S. aureus* culture filtrate; *Str*. CF, *S. pyogenes* culture filtrate; *Tri*. CF, *T. rubrum* culture filtrate.

significant effect on IL-8 secretion, indicating the protease inhibitors themselves would not inhibit IL-36γ activity (Figure 4B). Addition of the culture-filtrate-incubated samples to HaCaT cells revealed IL-36γ truncated in the presence of PMSF and EDTA both had IL-36γ-specific activity, while the addition of E64 prevented IL-36γ-mediated IL-8 secretion (Figure 4C). These results indicate a Cys protease secreted by *S. pyogenes* is responsible for the activation of IL-36γ.

During infection, *S. pyogenes* abundantly secretes the virulence factor and Cys protease SpeB. We therefore examined whether SpeB might also be responsible for the activation of IL-36γ by incubating SUMO-pro-IL-36γ with culture filtrate from a SpeB-deficient mutant strain of *S. pyogenes* (ΔSpeB). Analysis by SDS-PAGE showed that in contrast to wild-type *S. pyogenes*, IL-36γ did not undergo cleavage upon incubation with ΔSpeB *S. pyogenes* culture filtrate, and addition of the incubated sample to HaCaT cells did not elicit a strong IL-8 secretion (Figures 4D and 4E). These results suggest SpeB is responsible for processing of IL-36γ. For confirmation, SUMO-pro-IL-36γ was incubated with recombinant SpeB at 37°C for 1 h. Analysis by SDS-PAGE showed truncation of IL-36γ (Figure 4F). Furthermore, addition of SpeB and pro-IL-36γ to HaCaT cells resulted in secretion of IL-8 that could be inhibited by addition of IL-36RA (Figure 4G). Addition of SpeB or pro-IL-36γ alone resulted in little or no IL-8 secretion. These results therefore suggest IL-36γ is activated by SpeB secreted by *S. pyogenes.* Finally, the cleavage of pro-IL-36γ by SpeB was interrogated by LC-MS, which identified the prominent species with a mass corresponding to that of IL-36γ S18 (illustrated in Figure 4F).

To assess whether mIL-36γ is also susceptible to activation by SpeB, the above-mentioned experiments were repeated with recombinant pro-mIL-36γ. Cleavage assays showed that while wild-type *S. pyogenes* culture filtrate truncated mIL-36γ, ΔSpeB culture filtrate did not (Figure 4H). Furthermore, stimulation of MEFs with ΔSpeB culture-filtrate-incubated mIL-36γ did not elicit an IL-36-dependent response (Figure 4I). Finally, incubation of pro-mIL-36γ with recombinant SpeB resulted in rapid truncation of pro-mIL-36γ (within 5 min) that was identified by LC-MS as biologically active mature mIL-36γ (G13) (Figure 4J). Addition of SpeB-processed pro-mIL-36γ to MEFs also induced a strong response (Figure 4K). These results indicate both hIL-36γ and mIL-36γ are processed by SpeB to generate biologically active IL-36γ.

Aspergillus fumigatus Virulence Factor Asp F13 (Alp1) Activates Released IL-36γ in a Cell-Based Infection Assay

A similar approach was utilized to identify the activating protease produced by *A. fumigatus.* SDS-PAGE analysis of culture-filtrate-incubated SUMO-pro-IL-36γ indicated that cleavage

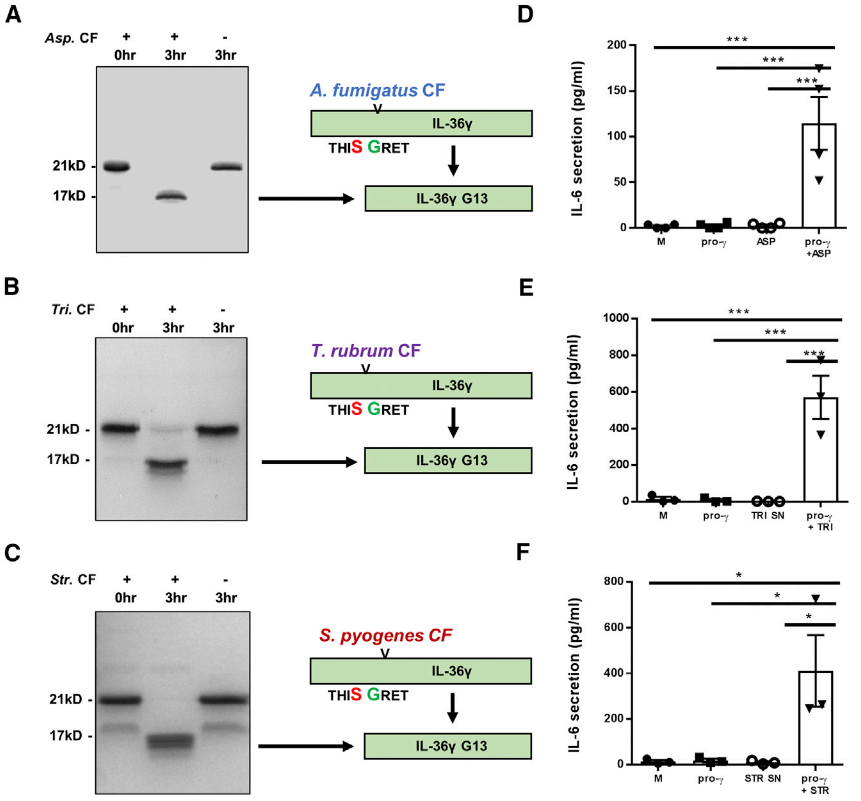

Figure 3. Mouse IL-36γ (mIL-36γ) Is Also Activated by Proteases Derived from a Range of Pathogens

(A–F) SUMO-tagged mIL-36γ (1 μg) was incubated at 37°C for 0 or 3 h, with or without 1 μL of either Asp. CF (A), *Tri.* CF (C), or *Str.* CF (E). Samples were analyzed by Coomassie-blue-stained SDS-PAGE gel. Cleaved products were also analyzed by mass spectrometry, with diagrams depicting the IL-36γ truncation generated in response to each CF. In addition, MEF cells (10^5 per well) were incubated for 24 h with M, pro-γ (10 nM), CF, or a combination of pro-γ and CF (B, D, and F). A one-way ANOVA was used to determine statistical significance of differences between treatment groups. *$p < 0.05$ and ***$p < 0.001$. Data shown are mean ± SEM (n = 3).

was unaffected when conducted in the presence of Cys protease inhibitor E64 and metalloproteinase inhibitor EDTA; however, the addition of Ser inhibitor PMSF completely ablated cleavage (Figure 5A). Testing these incubations for biological activity by addition to HaCaT cells reflected the results observed by SDS-PAGE. When added in combination with *Aspergillus* culture filtrate and pro-IL-36γ, the addition of E64 and EDTA had no effect on IL-36γ activity; however, the addition of PMSF significantly ablated IL-36γ-mediated IL-8 secretion (Figure 5B). These results implicate a Ser protease in the activation of IL-36γ.

As the secreted Ser protease Asp F13 has been identified as a major virulence factor secreted during infection, it provided a good candidate protease as an activator of IL-36γ. Therefore, culture filtrate from an Asp F13-deficient strain of *A. fumigatus* was examined against the wild type. As a negative control, a deficient strain of the virulence factor metalloprotease Asp F5 (Mep) was also examined. SUMO-pro-IL-36γ was incubated with wild-type, Asp F5$^{-/-}$, and Asp F13$^{-/-}$ *A. fumigatus* culture filtrate. SDS-PAGE resolution of the samples showed both wild-type and Asp F5$^{-/-}$ culture filtrates truncated IL-36γ, while Asp F13$^{-/-}$ did not (Figure 5C). Stimulation of HaCaT cells with wild-type and Asp F5$^{-/-}$ culture-filtrate-incubated pro-IL-36γ causes strong IL-8 secretion, while stimulation with Asp F13$^{-/-}$ culture-filtrate-incubated pro-IL-36γ had little effect on IL-8 secretion (Figure 5D). Finally, for confirmation, SUMO-IL-36γ was incubated with recombinant Asp F13 and Asp F5 and analyzed by SDS-PAGE and activity assay. Rapid cleavage was observed with recombinant Asp F13, but not Asp F5. Moreover, the addition of the incubated proteins to HaCaT cells revealed Asp F13-truncated IL-36γ-induced IL-8 secretion could be inhibited by addition of IL-36RA, whereas Asp F5-incubated pro-IL-36γ had no effect (Figures 5E and 5F). These results suggest IL-36γ is truncated and activated by *A. fumigatus* Ser protease Asp F13 (confirmed by MS; Figure S3).

We also tested whether Asp F13 and Asp F5 were capable of activating mIL-36γ. Recombinant pro-mIL-36γ was incubated with wild-type, Asp F13$^{-/-}$, and Asp F5$^{-/-}$ *Aspergillus* culture filtrate. SDS-PAGE analysis showed both wild-type and Asp F5$^{-/-}$ culture filtrate truncated pro-mIL-36γ, whereas Asp F13$^{-/-}$ did not (Figure 5G). Addition of the incubated proteins to MEFs showed that in contrast to wild-type- and Asp F5$^{-/-}$-culture-filtrate-incubated mIL-36γ, Asp F13$^{-/-}$-culture-filtrate-incubated pro-mIL-36γ had no biological activity (Figure 5I). Finally, as with hIL-36γ, mIL-36γ was incubated with recombinant Asp F13 and Asp F5. Analysis by SDS-PAGE showed that unlike Asp F5, Asp F13 rapidly processed mIL-36γ, and subsequent addition to MEFs revealed the Asp F13-processed mIL-36γ to be biologically active (Figures 5H and 5J). These results indicate Asp F13 processes both hIL-36γ and mIL-36γ to a biologically active mature cytokine.

Finally, after establishing that Asp F13 can activate recombinant IL-36γ, we utilized a cell-based infection assay to examine whether *Aspergillus* would also release and activate IL-36γ in an Asp F13-dependent fashion, or whether pathogen-mediated cell damage in the absence of Asp F13 is in itself enough to induce activation of IL-36γ. A stable HEK293 cell line expressing C-terminal FLAG-tagged pro-IL-36γ (proγF-293) was generated as outlined in the STAR Methods (Figure S4). The proγF-293 cells were inoculated with conidia of wild-type and mutant strains of *A. fumigatus* for 24 h, and the expressed IL-36γ in the supernatant was immunoprecipitated by its C-terminal FLAG tag. Western blot analysis showed IL-36γ was released into the supernatant by all strains of *Aspergillus*. Furthermore, supernatant from wild-type- and Asp F5$^{-/-}$-infected proγF-293 cells contained truncated IL-36γ, shown by activity assay to be biologically active (Figures 6A and 6B). Supernatant from cells infected

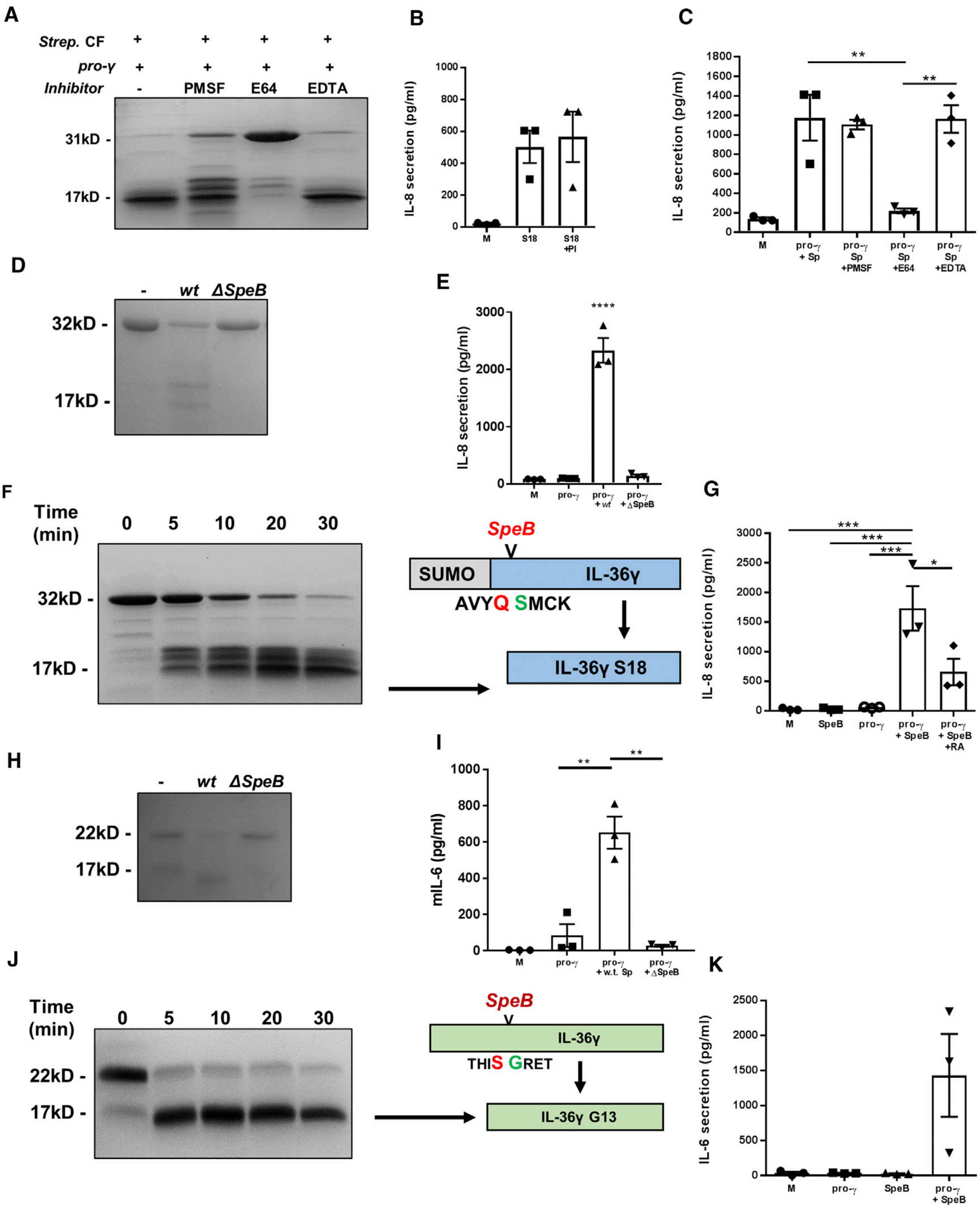

Figure 4. Activation of IL-36γ by *S. pyogenes* Is Dependent on SpeB

(A–K) (A) SUMO-tagged human IL-36γ (1 μg) was incubated at 37°C for 3 h with *Strep*. CF, with and without the Ser inhibitor PMSF (10 mM), the Cys protease inhibitor E64 (20 μM), or metalloproteinase inhibitor EDTA (5 mM). (B) HaCaT cells (105 per well) were incubated for 24 h with M or S18 (10 nM), with and without a broad-range protease inhibitor that includes PMSF, E64, and EDTA (PI). (C–K) HaCaT cells (10^5 per well) were incubated for 24 h with M, pro-γ, and *S. pyogenes* CF (Sp; 1 μL), pro-γ and *S. pyogenes* CF with PMSF (0.1 mM), E64 (0.2 μM), or EDTA. SUMO-tagged human IL-36γ (1 μg; D) or mouse pro-γ (H) was incubated at 37°C for 3 h with *wt* and ΔSpeB *S. pyogenes* CF. HaCaT cells (E) or MEFs (I) (10^5 per well) were incubated for 24 h with M, pro-γ (10 nM), or pro-γ and *wt* (*wt* Sp) or ΔSpeB *S. pyogenes* CF. HaCaT cells were stimulated with human pro-γ, while MEFs were stimulated with mouse pro-γ. SUMO-tagged human IL-36γ (1 μg; F) or mouse pro-γ (J) was incubated at 37°C with 20 ng of recombinant SpeB for 0–30 min. Cleaved products were also analyzed by mass spectrometry, with diagrams depicting the IL-36γ truncation generated. HaCaT cells (G) and MEFs (K) were stimulated with M, pro-γ, recombinant SpeB, or pro-γ with SpeB (and pro-γ +

(legend continued on next page)

with Asp F13$^{-/-}$ did contain pro-IL-36γ, indicating cell damage induced release of the cytokine; yet, IL-36γ remained in its pro-form and when tested by activity assay did not show any biological activity. These results indicate in the context of an infection, IL-36γ is released in its inactive form as a result of cell damage and then activated by Asp F13 secreted by *A. fumigatus* during infection.

DISCUSSION

The mechanisms surrounding IL-36γ release from cells are not completely understood. As leaderless proteins, the IL-36 cytokines do not enter the canonical secretory system following synthesis, and unlike IL-1β and IL-18, the underlying mechanisms of release are unclear. Indeed, in the majority of experimental systems examined, induced IL-36 cytokines appear to be retained as intracellular cytokines. This is also true of the IL-1 family alarmins IL-33 and IL-1α, which function as proinflammatory mediators following release as a result of cellular damage. In this study, we demonstrated that while microbial stimulation of epithelial cells increased intracellular stores of IL-36γ, the cytokine was only released when incubated with live pathogens. The live commensal *S. epidermidis* did not induce release of IL-36γ despite increasing its intracellular expression (Figure 1). Indeed, release of IL-36γ induced by pathogenic microbes paralleled that of the cytosolic housekeeping protein GAPDH, suggesting that in the context of infection the major mechanism of IL-36γ release may be a result of pathogen-induced membrane damage or necrosis rather than through an active response to microbial challenge, perhaps similarly to IL-33 and IL-1α. It therefore seems likely that known cytotoxic components such as pore-forming toxins secreted by the pathogens would be responsible for release of IL-36γ. The use of lytic factors to destroy host cells for nutrient acquisition and immune evasion is a common feature of many invasive pathogens, and both *S. pyogenes* and *A. fumigatus* produce cytotoxins such as streptolysin O and Asp-hemolysin, respectively, during infection (Singh et al., 2012; Nilsson et al., 2006; Wartenberg et al., 2011). It may therefore be likely that numerous pathogens will have the ability to release IL-36γ from epithelial cells. Furthermore, by incubating recombinant pro-IL-36γ with pathogen culture filtrates, we demonstrated that the epithelial pathogens *A. fumigatus*, *S. pyogenes*, *T. rubrum*, and *S. aureus* all produce proteases that process recombinant IL-36γ into its mature form (Figure 2), suggesting once released from epithelial tissue IL-36γ will undergo activation, as was demonstrated with *A. fumigatus* (Figure 6). Therefore, IL-36-mediated signaling is likely to occur shortly after infection-induced damage, initiating and orchestrating an immune response directed against extracellular pathogens. As the epithelial microbes tested spanned both fungi and bacteria associated with infection at different epithelial sites, and given the apical expression of IL-36γ, we believe these results indicate that IL-36γ is broadly sensitive to pathogen-derived proteases, and its release and activation following pathogen-mediated damage implicate IL-36γ as an epithelial alarmin.

The results were further supported by the fact that recombinant mIL-36γ was activated by the same culture filtrates (Figure 3). This was somewhat surprising as bioactive forms of hIL-36γ and mIL-36γ were generated by disparate classes of pathogen-derived proteases despite the requirement of precise cleavage (human Gln_{17}-Ser_{18}, mouse Tyr_{12}-Gly_{13}) and non-conserved primary amino acid sequences proximal to the cleavage sites. Structural information obtained from the crystal structure of hIL-36γ shows the N terminus to be a flexible, exposed region that protrudes from the compact core of the protein's IL-1 domain and would therefore be more accessible for processing (Günther and Sundberg, 2014). Structural alignment of multiple species suggests that despite the differences in primary amino acid sequences, the exposed flexible N terminus is a common feature; therefore, protease sensitivity is likely to be an evolutionarily conserved phenomenon. Furthermore, analogous protease sensitivity has been recently described for other IL-1 family members, most notably IL-33, shown to be sensitive to activation by numerous allergen proteases. Unlike IL-36γ, however, protease sensitivity of IL-33 triggers type II immune responses (Cayrol et al., 2018). Additionally, while the other IL-36 cytokines were not extensively tested in this study, we did demonstrate that IL-36α also undergoes activation following incubation with *A. fumigatus* and *T. rubrum* culture filtrate (Figure S5). Having three functionally similar cytokines with structurally distinct cleavage sites may prove advantageous in the detection of invasive pathogens by enabling sensitivity to a wider range of pathogens. It has also been proposed that the recent evolution of multiple IL-36 genes from an ancestral IL-1 gene could provide a system that would resist microbial immune evasion (Jensen, 2017). Indeed, multiple pathogens, particularly DNA viruses, are known to prevent IL-1 activity via cytokine binding proteins, increased cytokine degradation, and inhibition of cytokine-activating proteases (Richards et al., 2014). The existence of three IL-36 agonists would therefore circumvent similar immune evasion strategies (Jensen, 2017).

Identifying virulence factors secreted by pathogens as activators of IL-36γ has implications for the role microbial infection may have in inflammatory conditions. As several non-infectious inflammatory conditions have microbial etiologies, these observations may provide a mechanism for the initiation of inflammation via microbe-induced IL-36-mediated signaling. In the clinical setting, it is well established that streptococcal throat infection is the major triggering factor of guttate psoriasis and that IL-36γ is abundantly expressed in the outermost skin layers of psoriatic individuals (D'Erme et al., 2015; Telfer et al., 1992). In some patients the connection between streptococcal infection and psoriasis flare is so strong that guidelines recommend tonsillectomy (Simões et al., 2015). As streptococcal SpeB is the most highly secreted protein produced during infection and is known to interact with host proteins with both pro- and anti-inflammatory

SpeB with IL-36RA; G). HaCaT cells were stimulated with human IL-36γ, while MEFs were stimulated with mIL-36γ. Samples were analyzed by Coomassie-blue-stained SDS-PAGE gel (A, D, F, H, and J) or measured by ELISA (B, C, E, G, I, and K). A one-way ANOVA was used to determine statistical significance of differences between treatment groups. *p < 0.05, **p < 0.01, and ***p < 0.001. Data shown are mean ± SEM (n = 3). Abbreviations are as follows: S18, IL-36γ S18; *Strep*. CF, *S. pyogenes* culture filtrate; *wt*, wild type.

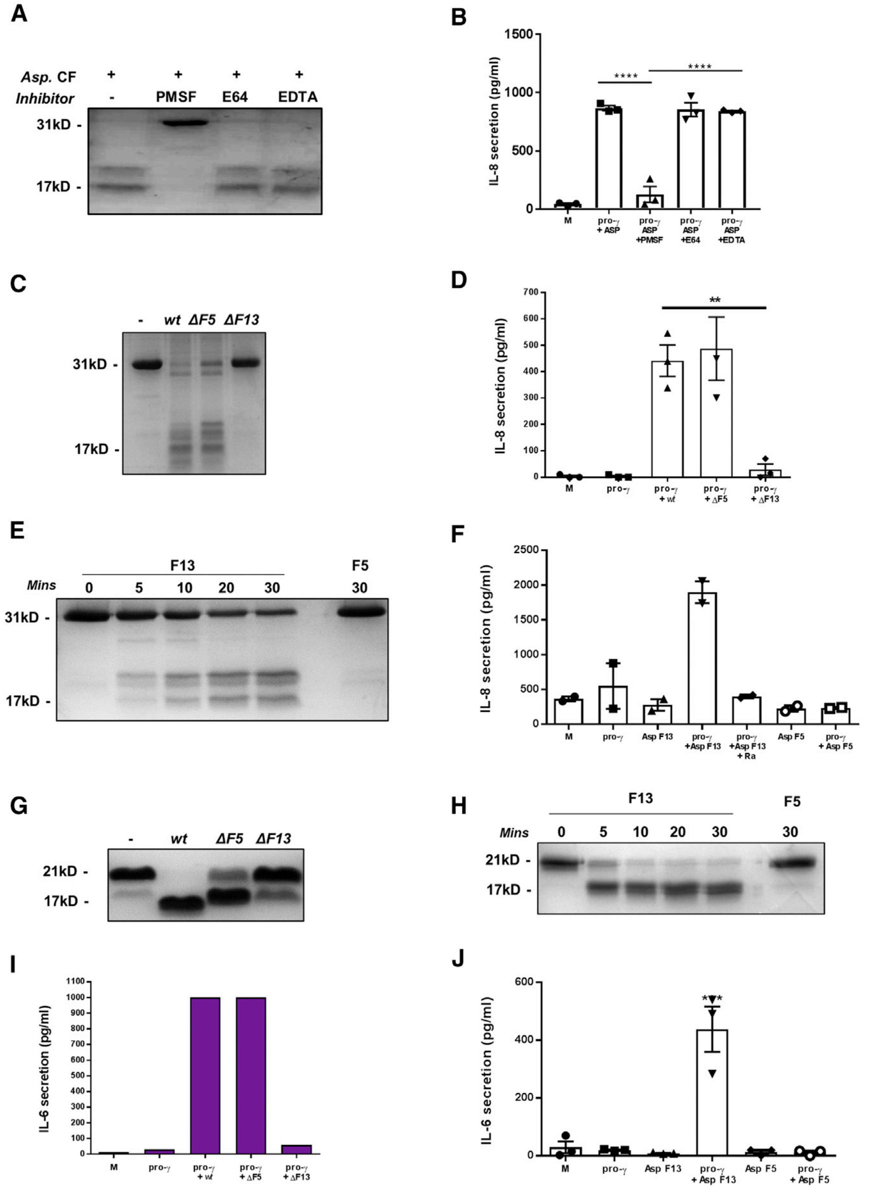

Figure 5. Activation of IL-36γ by *Aspergillus* Is Dependent on the Virulence Factor Asp F13

(A–J) (A) SUMO-tagged human IL-36γ (1 μg) was incubated at 37°C for 3 h with *Asp*. CF, with and without the Ser inhibitor PMSF (10 mM), the Cys protease inhibitor E64 (20 μM), or metalloproteinase inhibitor EDTA (5 mM). (B–J) HaCaT cells (10^5 per well) were incubated for 24 h with M, or pro-γ (10 nM) and *Asp*. CF filtrate (1 μL), with and without PMSF (0.1 mM), E64 (0.2 μM), or EDTA. SUMO-tagged human IL-36γ (1 μg; C) or mouse pro-γ (G) was incubated at 37°C 3 h with *wt*, ΔF5, or ΔF13 *Asp.* CF. HaCaT cells (D) or MEFs (I) (10^5 per well) were incubated for 24 h with M, pro-γ (10 nM), or pro-γ and *wt*, ΔF5, or ΔF13 *Asp*. CF. HaCaT cells were stimulated with human pro-γ, while MEFs were stimulated with mouse pro-γ. SUMO-tagged human IL-36γ (1 μg; E) or mouse pro-γ (H) was incubated at 37°C with 20 ng of recombinant F13 or F5 for 0–30 min. HaCaT cells (F) and MEFs (J) were stimulated with M, pro-γ, recombinant F13, recombinant F5, pro-γ with F13, or pro-γ with F5. HaCaT cells were stimulated with human IL-36γ, while MEFs were stimulated with mIL-36γ. Samples were analyzed by Coomassie-blue-stained SDS-PAGE gel (A, C, E, G, and H) or measured by ELISA (B, D, F, I, and J). A one-way ANOVA was used to determine statistical significance of differences between treatment groups. **$p < 0.01$, ***$p < 0.001$, and ****$p < 0.0001$. Data shown are mean ± SEM (B, D, and J, n = 3; F, n = 2; I, n = 1). Abbreviations are as follows: ΔF5, Asp $F5^{-/-}$; ΔF13, Asp $F13^{-/-}$; F5, Asp F5; F13, Asp F13.

consequences, the data presented here may well provide a mechanistic link between the proteolytic activation of IL-36γ by *S. pyogenes* SpeB and the initiation of guttate psoriasis (Nelson et al., 2011; Elliott, 1945). Published data have also shown that IL-36γ is expressed in other inflammatory skin diseases associated with microbial infection including hydradenitis suppurative, eczema, and tinea, suggesting a potential causative role (Di Caprio et al., 2017; Hessam et al., 2018; Otobe et al., 2018). IL-36γ is also upregulated in inflamed intestinal tissue as a result of stimulation from microbiota (Russell et al., 2016; Nishida et al., 2016). With a heavy microbial presence in the gut and increased expression of IL-36 cytokines, it seems plausible tissue damage and production of microbial proteases in inflammatory bowel disease might facilitate initiation of inflammation through liberation and activation of IL-36 cytokines. In addition to its pathological role, IL-36 signaling in the gut has been demonstrated to be critical in both barrier defense and wound repair (Ngo et al., 2018). IL-36 signaling is a potent inducer of IL-23—a cytokine that, in turn, is a significant inducer of IL-22—providing a bridge between immune activation and epithelial repair. Although not examined in this study, the protease sensitive nature of IL-36 cytokines also provides a potential sensing mechanism for gut parasites such as helminths. These parasites are well documented to secrete a variety of proteases and cause significant epithelial damage (Caffrey et al., 2018; McKay et al., 2017). The proteolytic activation of IL-36γ in the gut may therefore provide a potential mechanism to facilitate an inflammatory response and subsequent wound repair.

Epithelial barriers such as the skin, lungs, and gut perform multiple essential physiological functions and represent critical interfaces between an organism and its environment. However, due to the ubiquitous nature of commensal microbes and potentially damaging pathogens, these sites must be able to mount robust immune responses toward pathogens, while at the same time prevent detrimental inflammatory responses toward harmless commensals. The results obtained in this study have

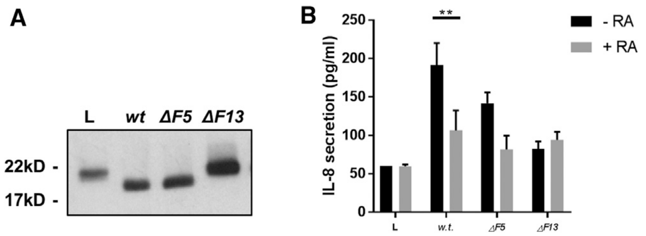

Figure 6. Human Pathogen *A. fumigatus* Induces the Release and Activation of Endogenous IL-36γ

(A) The proγF-293 cells were inoculated with *wt*, ΔF5, or ΔF13 *A. fumigatus* conidia for 24 h. Supernatants were removed from cells, and FLAG-tag-immunoprecipitated IL-36γ was analyzed by western blot alongside proγF-293 lysate (L).

(B) HaCaT cells were stimulated with 50 μL of proγF-293 supernatant after 24 h incubation following mechanical lysis (L), inoculation with *wt*, ΔF5, ΔF13 *A. fumigatus* conidia in the presence or absence of 1 μg/mL IL-36RA (RA). Harvested supernatants were tested for IL-8 concentration by ELISA. A one-way ANOVA was used to determine statistical significance of differences between treatment groups. $^{*}p < 0.05$ and $^{**}p < 0.01$. Data shown are mean ± SEM (n = 4).

demonstrated a scenario in which IL-36γ is induced, released from cells via pathogenic damage, and subsequently activated as a direct result of secreted proteolytic virulence factors by invasive epithelial pathogens. Given the characteristics of IL-36γ as an apically located epithelial initiator of inflammation and its sensitivity to pathogen-derived proteases, we believe this work demonstrates that IL-36γ functions as a global epithelial alarmin and broad sensor of pathogenic infection. This provides a critical mechanism whereby host organisms can discriminate invasive pathogens from harmless microbes. In addition to describing a general sensor of pathogenic presence, this work illustrates the potential for microbial infection to act as a trigger for IL-36-mediated pathological inflammation in susceptible individuals and helps build a more complete picture of how IL-36-mediated signaling is actually initiated in the context of epithelial infection, giving us a greater understanding of immune defenses at epithelial barriers.

STAR★METHODS

Detailed methods are provided in the online version of this paper and include the following:

- KEY RESOURCES TABLE
- RESOURCE AVAILABILITY
  - Lead Contact
  - Materials Availability
  - Data and Code Availability
- EXPERIMENTAL MODEL AND SUBJECT DETAILS
  - Cell lines
  - Microbial strains and culture conditions
- METHOD DETAILS
  - Reagents
  - Generation of recombinant proteins
  - Activity assays
  - Culture filtrate production
  - Infection assays
  - Immunohistochemistry
  - ELISA
  - FLAG Immunoprecipitation
  - LC-MS
  - Gel electrophoresis and western blotting
- QUANTIFICATION AND STATISTICAL ANALYSIS

SUPPLEMENTAL INFORMATION

Supplemental Information can be found online at https://doi.org/10.1016/j.celrep.2020.108515.

ACKNOWLEDGMENTS

This work was supported by the Fraunhofer Cluster of Excellence Immune-Mediated Diseases (CIMD) and Fraunhofer International Consortium for Anti-Infective Research (iCAIR). We thank D. Jonigk and G. Warnecke (Medical School, Germany) and L. Wilkens and H.-G. Fieguth (KRH Clinics Hannover, Germany) for pathological support and collaboration in lung tissue supply. This article also presents independent research supported by the National Institute for Health Research (NIHR) Leeds Biomedical Research Centre (BRC). The views expressed are those of the author(s) and not necessarily those of the NHS, the NIHR, or the Department of Health. The authors gratefully acknowledge the Medical Research Council (MRC) (MR/M01942X/1) and British Skin Foundation (044_R_19) for funding.

AUTHOR CONTRIBUTIONS

T.M., J.S.A., C.H., S.K., and A.B. undertook experimental work. M.S. and M.W. contributed equally to study design and supervision. P.B., Y.T., and S.H. provided vital reagents. All authors contributed to writing the manuscript, with T.M. taking the leading role.

DECLARATION OF INTERESTS

The authors declare no competing interests.

Received: February 14, 2020
Revised: October 5, 2020
Accepted: November 19, 2020
Published: December 15, 2020

REFERENCES

Ainscough, J.S., Macleod, T., McGonagle, D., Brakefield, R., Baron, J.M., Alase, A., Wittmann, M., and Stacey, M. (2017). Cathepsin S is the major activator of the psoriasis-associated proinflammatory cytokine IL-36γ. Proc. Natl. Acad. Sci. USA *114*, E2748–E2757.

Berekmeri, A., Latzko, A., Alase, A., Macleod, T., Ainscough, J.S., Laws, P., Goodfield, M., Wright, A., Helliwell, P., Edward, S., et al. (2018). Detection of IL-36gamma through noninvasive tape stripping reliably discriminates psoriasis from atopic eczema. J. Allergy Clin. Immunol. *142*, 988–991.e4.

Bianchi, M.E., Crippa, M.P., Manfredi, A.A., Mezzapelle, R., Rovere Querini, P., and Venereau, E. (2017). High-mobility group box 1 protein orchestrates responses to tissue damage via inflammation, innate and adaptive immunity, and tissue repair. Immunol. Rev. *280*, 74–82.

Boutet, M.A., Bart, G., Penhoat, M., Amiaud, J., Brulin, B., Charrier, C., Morel, F., Lecron, J.C., Rolli-Derkinderen, M., Bourreille, A., et al. (2016). Distinct expression of interleukin (IL)-36α, β and γ, their antagonist IL-36Ra and IL-38 in psoriasis, rheumatoid arthritis and Crohn's disease. Clin. Exp. Immunol. *184*, 159–173.

Braegelmann, J., Braegelmann, C., Bieber, T., and Wenzel, J. (2018). Candida induces the expression of IL-36γ in human keratinocytes: implications for a

CellPress
OPEN ACCESS

pathogen-driven exacerbation of psoriasis? J. Eur. Acad. Dermatol. Venereol. *32*, e403–e406.

Bridgewood, C., Fearnley, G.W., Berekmeri, A., Laws, P., Macleod, T., Ponnambalam, S., Stacey, M., Graham, A., and Wittmann, M. (2018). IL-36γ Is a Strong Inducer of IL-23 in Psoriatic Cells and Activates Angiogenesis. Front. Immunol. *9*, 200.

Burns, E.H., Jr., Marciel, A.M., and Musser, J.M. (1996). Activation of a 66-kilodalton human endothelial cell matrix metalloprotease by Streptococcus pyogenes extracellular cysteine protease. Infect. Immun. *64*, 4744–4750.

Caffrey, C.R., Goupil, L., Rebello, K.M., Dalton, J.P., and Smith, D. (2018). Cysteine proteases as digestive enzymes in parasitic helminths. PLoS Negl. Trop. Dis. *12*, e0005840.

Carrier, Y., Ma, H.L., Ramon, H.E., Napierata, L., Small, C., O'Toole, M., Young, D.A., Fouser, L.A., Nickerson-Nutter, C., Collins, M., et al. (2011). Inter-regulation of Th17 cytokines and the IL-36 cytokines in vitro and in vivo: implications in psoriasis pathogenesis. J. Invest. Dermatol. *131*, 2428–2437.

Cayrol, C., Duval, A., Schmitt, P., Roga, S., Camus, M., Stella, A., Burlet-Schiltz, O., Gonzalez-de-Peredo, A., and Girard, J.P. (2018). Environmental allergens induce allergic inflammation through proteolytic maturation of IL-33. Nat. Immunol. *19*, 375–385.

D'Erme, A.M., Wilsmann-Theis, D., Wagenpfeil, J., Hölzel, M., Ferring-Schmitt, S., Sternberg, S., Wittmann, M., Peters, B., Bosio, A., Bieber, T., and Wenzel, J. (2015). IL-36γ (IL-1F9) is a biomarker for psoriasis skin lesions. J. Invest. Dermatol. *135*, 1025–1032.

Di Caprio, R., Balato, A., Caiazzo, G., Lembo, S., Raimondo, A., Fabbrocini, G., and Monfrecola, G. (2017). IL-36 cytokines are increased in acne and hidradenitis suppurativa. Arch. Dermatol. Res. *309*, 673–678.

Dunn, E., Sims, J.E., Nicklin, M.J., and O'Neill, L.A. (2001). Annotating genes with potential roles in the immune system: six new members of the IL-1 family. Trends Immunol. *22*, 533–536.

Eigenbrod, T., Park, J.H., Harder, J., Iwakura, Y., and Núñez, G. (2008). Cutting edge: critical role for mesothelial cells in necrosis-induced inflammation through the recognition of IL-1 alpha released from dying cells. J. Immunol. *181*, 8194–8198.

Elliott, S.D. (1945). A Proteolytic Enzyme Produced by Group a Streptococci with Special Reference to Its Effect on the Type-Specific M Antigen. J. Exp. Med. *81*, 573–592.

Foster, A.M., Baliwag, J., Chen, C.S., Guzman, A.M., Stoll, S.W., Gudjonsson, J.E., Ward, N.L., and Johnston, A. (2014). IL-36 promotes myeloid cell infiltration, activation, and inflammatory activity in skin. J. Immunol. *192*, 6053–6061.

Gabay, C., and Towne, J.E. (2015). Regulation and function of interleukin-36 cytokines in homeostasis and pathological conditions. J. Leukoc. Biol. *97*, 645–652.

Gao, N., Me, R., Dai, C., Seyoum, B., and Yu, F.X. (2018). Opposing Effects of IL-1Ra and IL-36Ra on Innate Immune Response to *Pseudomonas aeruginosa* Infection in C57BL/6 Mouse Corneas. J. Immunol. *201*, 688–699.

Gresnigt, M.S., Rösler, B., Jacobs, C.W., Becker, K.L., Joosten, L.A., van der Meer, J.W., Netea, M.G., Dinarello, C.A., and van de Veerdonk, F.L. (2013). The IL-36 receptor pathway regulates Aspergillus fumigatus-induced Th1 and Th17 responses. Eur. J. Immunol. *43*, 416–426.

Günther, S., and Sundberg, E.J. (2014). Molecular determinants of agonist and antagonist signaling through the IL-36 receptor. J. Immunol. *193*, 921–930.

He, Q., Chen, H.X., Li, W., Wu, Y., Chen, S.J., Yue, Q., Xiao, M., and Li, J.W. (2013). IL-36 cytokine expression and its relationship with p38 MAPK and NF-κB pathways in psoriasis vulgaris skin lesions. J. Huazhong Univ. Sci. Technolog. Med. Sci. *33*, 594–599.

Henry, C.M., Sullivan, G.P., Clancy, D.M., Afonina, I.S., Kulms, D., and Martin, S.J. (2016). Neutrophil-Derived Proteases Escalate Inflammation through Activation of IL-36 Family Cytokines. Cell Rep. *14*, 708–722.

Hessam, S., Sand, M., Gambichler, T., Skrygan, M., Rüddel, I., and Bechara, F.G. (2018). Interleukin-36 in hidradenitis suppurativa: evidence for a distinctive proinflammatory role and a key factor in the development of an inflammatory loop. Br. J. Dermatol. *178*, 761–767.

Iadarola, P., Lungarella, G., Martorana, P.A., Viglio, S., Guglielminetti, M., Korzus, E., Gorrini, M., Cavarra, E., Rossi, A., Travis, J., and Luisetti, M. (1998). Lung injury and degradation of extracellular matrix components by Aspergillus fumigatus serine proteinase. Exp. Lung Res. *24*, 233–251.

Jensen, L.E. (2017). Interleukin-36 cytokines may overcome microbial immune evasion strategies that inhibit interleukin-1 family signaling. Sci. Signal. *10*, eaan3589.

Johnston, A., Xing, X., Guzman, A.M., Riblett, M., Loyd, C.M., Ward, N.L., Wohn, C., Prens, E.P., Wang, F., Maier, L.E., et al. (2011). IL-1F5, -F6, -F8, and -F9: a novel IL-1 family signaling system that is active in psoriasis and promotes keratinocyte antimicrobial peptide expression. J. Immunol. *186*, 2613–2622.

Kono, H., Karmarkar, D., Iwakura, Y., and Rock, K.L. (2010). Identification of the cellular sensor that stimulates the inflammatory response to sterile cell death. J. Immunol. *184*, 4470–4478.

Kovach, M.A., Singer, B., Martinez-Colon, G., Newstead, M.W., Zeng, X., Mancuso, P., Moore, T.A., Kunkel, S.L., Peters-Golden, M., Moore, B.B., and Standiford, T.J. (2017). IL-36γ is a crucial proximal component of protective type-1-mediated lung mucosal immunity in Gram-positive and -negative bacterial pneumonia. Mucosal Immunol. *10*, 1320–1334.

Lin, S.J., Schranz, J., and Teutsch, S.M. (2001). Aspergillosis case-fatality rate: systematic review of the literature. Clin. Infect. Dis. *32*, 358–366.

Macleod, T., Doble, R., McGonagle, D., Wasson, C.W., Alase, A., Stacey, M., and Wittmann, M. (2016). Neutrophil Elastase-mediated proteolysis activates the anti-inflammatory cytokine IL-36 Receptor antagonist. Sci. Rep. *6*, 24880.

McKay, D.M., Shute, A., and Lopes, F. (2017). Helminths and intestinal barrier function. Tissue Barriers *5*, e1283385.

Namvar, S., Warn, P., Farnell, E., Bromley, M., Fraczek, M., Bowyer, P., and Herrick, S. (2015). Aspergillus fumigatus proteases, Asp f 5 and Asp f 13, are essential for airway inflammation and remodelling in a murine inhalation model. Clin. Exp. Allergy *45*, 982–993.

Nelson, D.C., Garbe, J., and Collin, M. (2011). Cysteine proteinase SpeB from Streptococcus pyogenes - a potent modifier of immunologically important host and bacterial proteins. Biol. Chem. *392*, 1077–1088.

Ngo, V.L., Abo, H., Maxim, E., Harusato, A., Geem, D., Medina-Contreras, O., Merlin, D., Gewirtz, A.T., Nusrat, A., and Denning, T.L. (2018). A cytokine network involving IL-36γ, IL-23, and IL-22 promotes antimicrobial defense and recovery from intestinal barrier damage. Proc. Natl. Acad. Sci. USA *115*, E5076–E5085.

Nilsson, M., Sørensen, O.E., Mörgelin, M., Weineisen, M., Sjöbring, U., and Herwald, H. (2006). Activation of human polymorphonuclear neutrophils by streptolysin O from Streptococcus pyogenes leads to the release of proinflammatory mediators. Thromb. Haemost. *95*, 982–990.

Nishida, A., Hidaka, K., Kanda, T., Imaeda, H., Shioya, M., Inatomi, O., Bamba, S., Kitoh, K., Sugimoto, M., and Andoh, A. (2016). Increased Expression of Interleukin-36, a Member of the Interleukin-1 Cytokine Family, in Inflammatory Bowel Disease. Inflamm. Bowel Dis. *22*, 303–314.

Oppenheim, J.J., and Yang, D. (2005). Alarmins: chemotactic activators of immune responses. Curr. Opin. Immunol. *17*, 359–365.

Otobe, S., Sugaya, M., Nakajima, R., Oka, T., Takahashi, N., Kabasawa, M., Miyagaki, T., Asano, Y., and Sato, S. (2018). Increased interleukin-36γ expression in skin and sera of patients with atopic dermatitis and mycosis fungoides/Sézary syndrome. J. Dermatol. *45*, 468–471.

Ramadas, R.A., Ewart, S.L., Medoff, B.D., and LeVine, A.M. (2011). Interleukin-1 family member 9 stimulates chemokine production and neutrophil influx in mouse lungs. Am. J. Respir. Cell Mol. Biol. *44*, 134–145.

Richards, K.H., Doble, R., Wasson, C.W., Haider, M., Blair, G.E., Wittmann, M., and Macdonald, A. (2014). Human papillomavirus E7 oncoprotein increases production of the anti-inflammatory interleukin-18 binding protein in keratinocytes. J. Virol. *88*, 4173–4179.

Russell, S.E., Horan, R.M., Stefanska, A.M., Carey, A., Leon, G., Aguilera, M., Statovci, D., Moran, T., Fallon, P.G., Shanahan, F., et al. (2016). IL-36α

expression is elevated in ulcerative colitis and promotes colonic inflammation. Mucosal Immunol. *9*, 1193–1204.

Simões, J.F., Ribeiro, J., Ferreira, B.R., and Paiva, S. (2015). The role of tonsillectomy in psoriasis treatment. BMJ Case Rep. *2015*, bcr2014206899.

Singh, B., Fleury, C., Jalalvand, F., and Riesbeck, K. (2012). Human pathogens utilize host extracellular matrix proteins laminin and collagen for adhesion and invasion of the host. FEMS Microbiol. Rev. *36*, 1122–1180.

Tamura, F., Nakagawa, R., Akuta, T., Okamoto, S., Hamada, S., Maeda, H., Kawabata, S., and Akaike, T. (2004). Proapoptotic effect of proteolytic activation of matrix metalloproteinases by Streptococcus pyogenes thiol proteinase (Streptococcus pyrogenic exotoxin B). Infect. Immun. *72*, 4836–4847.

Telfer, N.R., Chalmers, R.J., Whale, K., and Colman, G. (1992). The role of streptococcal infection in the initiation of guttate psoriasis. Arch. Dermatol. *128*, 39–42.

Temann, A., Golovina, T., Neuhaus, V., Thompson, C., Chichester, J.A., Braun, A., and Yusibov, V. (2017). Evaluation of inflammatory and immune responses in long-term cultured human precision-cut lung slices. Hum. Vaccin. Immunother. *13*, 351–358.

Terao, Y., Mori, Y., Yamaguchi, M., Shimizu, Y., Ooe, K., Hamada, S., and Kawabata, S. (2008). Group A streptococcal cysteine protease degrades C3 (C3b) and contributes to evasion of innate immunity. J. Biol. Chem. *283*, 6253–6260.

Towne, J.E., Renshaw, B.R., Douangpanya, J., Lipsky, B.P., Shen, M., Gabel, C.A., and Sims, J.E. (2011). Interleukin-36 (IL-36) ligands require processing for full agonist (IL-36α, IL-36β, and IL-36γ) or antagonist (IL-36Ra) activity. J. Biol. Chem. *286*, 42594–42602.

Verma, A.H., Zafar, H., Ponde, N.O., Hepworth, O.W., Sihra, D., Aggor, F.E.Y., Ainscough, J.S., Ho, J., Richardson, J.P., Coleman, B.M., et al. (2018). IL-36 and IL-1/IL-17 Drive Immunity to Oral Candidiasis via Parallel Mechanisms. J. Immunol. *201*, 627–634.

Wartenberg, D., Lapp, K., Jacobsen, I.D., Dahse, H.M., Kniemeyer, O., Heinekamp, T., and Brakhage, A.A. (2011). Secretome analysis of Aspergillus fumigatus reveals Asp-hemolysin as a major secreted protein. Int. J. Med. Microbiol. *301*, 602–611.

Yang, D., Han, Z., and Oppenheim, J.J. (2017). Alarmins and immunity. Immunol. Rev. *280*, 41–56.

CellPress
OPEN ACCESS

STAR★METHODS

KEY RESOURCES TABLE

REAGENT or RESOURCE	SOURCE	IDENTIFIER
Antibodies		
Polyclonal goat anti-IL-36γ antibody	R and D Systems	Cat# BAF2320; RRID:AB_2280258
Monoclonal mouse anti-FLAG M2 antibody	Sigma-Aldrich	Cat# F3165; RRID:AB_259529
Bacterial and Virus Strains		
BL21-CodonPlus (DE3)-RIL *E. coli*	Agilent	Cat# 230245
Biological Samples		NA
Chemicals, Peptides, and Recombinant Proteins		
Human IL-36γ	Ainscough et al., 2017; This lab	PMID: 28289191
Human IL-36γ S18	Ainscough et al., 2017; This lab	PMID: 28289191
Human IL-36α	Ainscough et al., 2017; This lab	PMID: 28289191
Human IL-36RA V2	Ainscough et al., 2017; This lab	PMID: 28289191
Mouse IL-36γ	This paper	NA
Mouse IL-36γ G13	This paper	NA
Mouse IL-36α	This paper	NA
Protease Inhibitor Cocktail	ThermoFischer	Cat# A32955
PMSF	Merck Millipore	Cat# 52332
E64	Merck Millipore	Cat# 324890
Critical Commercial Assays		
IL-8 ELISA	BioLegend	431504
IL-36γ ELISA	Berekmeri et al., 2018; This lab	PMID: 29782895
Deposited Data		
Experimental Models: Cell Lines		
TR146	ECACC	Cat# 10032305, RRID:CVCL_2736
HEK293T	ATCC	Cat# CRL-3216, RRID:CVCL_0063
HaCaT	CLS	Cat# 300493/p800_HaCaT, RRID:CVCL_0038
proγF-293	This paper	NA
Experimental Models: Organisms/Strains		
Streptococcus pyogenes	Terao et al., 2008; Provided by Prof. Yutaka Terao, Niigata University	PMID: 18160402
Streptococcus pyogenes Δ*speB*	Terao et al., 2008; Provided by Prof. Yutaka Terao, Niigata University	PMID: 18160402
Staphylococcus aureus	ATCC	Cat# 6534
Staphylococcus epidermidis	ATCC	Cat# 12228
Aspergillus fumigatus	Namvar et al., 2015; Provided by Dr Sarah Herrick, University of Manchester	PMID: 25270353
Aspergillus fumigatus Asp F5$^{-/-}$	Namvar et al., 2015; Provided by Dr Sarah Herrick, University of Manchester	PMID: 25270353
Aspergillus fumigatus Asp F13$^{-/-}$	Namvar et al., 2015; Provided by Dr Sarah Herrick, University of Manchester	PMID: 25270353
Trichophyton rubrum	ATCC	Cat# 28188
Software and Algorithms		
GraphPad Prizm 7	GraphPad	https://www.graphpad.com

RESOURCE AVAILABILITY

Lead Contact

Further information and requests for resources and reagents should be directed to and will be fulfilled by the Lead Contact, Martin Stacey (M.Stacey@leeds.ac.uk).

Materials Availability

All proteins and cell lines generated and used in this study are available on request from the Lead Contact, Martin Stacey.

Data and Code Availability

This study did not generate or analyze datasets or code.

EXPERIMENTAL MODEL AND SUBJECT DETAILS

Cell lines

TR146, HEK293T, and HaCaT cells were used in this study. The cell line proγF-293 was also generated from HEK293 cells in the following method. cDNA of pro-IL-36γ was cloned into pcDNA3.1 vector containing a G418 resistance gene using reverse primers containing a C-terminal FLAG tag to generate the fusion protein pro-IL-36γ -FLAG. The vector was linearized prior to transfection into HEK293 cells and stable transfectants were selected using G418 (500 μg/ml). Stable transfectants were subject to limiting dilution to generate monoclonal colonies, which were bulked and screened for strong IL-36γ expression by ELISA. Successful production of FLAG-tagged IL-36γ was confirmed by western blot (Figure S1).

All cells were cultured in FCS-supplemented culture medium (DMEM; Life technologies), containing 400 μg/ml penicillin/streptomycin and 10% FCS (Life Technologies) at 37°C in a 5% CO_2 incubator.

Microbial strains and culture conditions

A wild-type *S. pyogenes* strain and its isogenic Δ*speB* mutant were isolated and generated as previously described (Terao et al., 2008). *S. pyogenes* were cultured in Todd-Hewitt broth supplemented with 0.5% yeast extract (THY). *S. aureus* and *S. epidermidis* were cultured in 2YT media. *A. fumigatus* and its Asp F 13 and Asp F 5 mutants were generated as previously described (Namvar et al., 2015). *A. fumigatus* and *T. rubrum* were cultured with Sabouraud dextrose or agar. Conidia were harvested from agar by washing with 0.05% Tween-20 in PBS.

METHOD DETAILS

Reagents

The protease inhibitors PMSF and E64 were purchased from Merck Millipore. Total protease inhibitor cocktail was obtained from Thermo Fischer. For Western-blot analysis, the primary antibodies used were a biotinylated goat anti-IL-36γ antibody and mouse anti-FLAG M2. HRP-conjugated avidin and HRP-conjugated anti-mouse were used for detection.

Generation of recombinant proteins

cDNA of full length, pro human and mouse IL-36γ, IL-36α, IL-36RA V2, human IL-36γ S18 and mouse IL-36γ G13 were cloned into a Champion pET SUMO expression vector (Invitrogen, UK). Proteins were expressed in BL21-CodonPlus (DE3)-RIL *E. coli* overnight at 25°C and soluble proteins purified via Ni^{2+}-affinity and size exclusion chromatography. Proteins were further purified by Ni^{2+}-affinity chromatography prior to overnight cleavage of N-terminal SUMO by the Ulp1 protease, followed by subsequent ion exchange and size exclusion chromatography into 20 mM Tris pH7.4, 300 mM NaCl as previously described (Macleod et al., 2016; Ainscough et al., 2017).

Activity assays

Activity assays were performed as previously described (Ainscough et al., 2017). Briefly, HaCaT cells were plated at $2x10^5$ cells/well (24-well plate) in complete culture media, and cultured to 90% confluence. The media was then removed and replaced with fresh media. For most activity assay experiments, indicated treatments were added and cells incubated for 24 hours at 37°C. For activity assay experiments involving protease inhibitors, samples were preincubated with protease inhibitors for 3 hours at 4°C before being added to HaCaT cells. For the activity assay experiments involving cell supernatants from treated proγF-293 cells, supernatants were centrifuged for removal of microbial debris and preincubated with total protease inhibitors for 3 hours at 4°C prior to addition to HaCaT cells. Following incubation, cell supernatants were removed and frozen at −80°C.

Culture filtrate production

5 mL of appropriate growth medium was inoculated with microbes and incubated at 37°C for 24 hours. Cultures were then centrifuged, and supernatants were removed and filtered.

Infection assays

proγF-293 s were plated in 6 well plates and grown to 90% confluence. Cells were washed 2x in PBS and media replaced with infection assay medium (PBS, 1 g/L dextrose, 100 mg/L $MgCl_2$, 100 mg/L $CaCl_2$, 30 mM HEPES). Cells were inoculated with the indicated amount of pathogen and incubated at 37°C 5% CO_2 for the time indicated. Supernatants were removed and treated according to FLAG immunoprecipitation or activity assay.

Immunohistochemistry

Formalin-fixed, paraffin-embedded skin sections were stained using standard hematoxylin and eosin, as well as periodic acid Schiff staining. IL-36γ protein expression was analyzed by IHC using the monoclonal mouse IgG1 anti-human-IL-36γ antibody ab156783 (Abcam Inc., Cambridge, MA) without pretreatment with a dilution of 1:500. Visualization was performed using the REAL staining kit (DAKO, Hamburg, Germany) with Fast Red as chromogen (Braegelmann et al., 2018).

ELISA

Supernatants were analyzed for IL-8 protein using a specific ELISA kit from Biolegend (San Diego, CA). Supernatants and lysates were analyzed for IL-36γ protein using an in-house monoclonal based ELISA previously described (Berekmeri et al., 2018). IL-8 ELISA was performed following the manufacturer's instructions. IL-36γ ELISA was performed as follows. Immunosorbent 96-well ELISA plates were coated with 2 μg/mL capture antibody in PBS at 4°C overnight. Plates were then washed with 0.1% Tween 20/PBS and blocked for 1 hour in 2% BSA in 0.1% Tween-20/PBS. Samples were incubated subsequently for 1 hour at room temperature before washing and incubation with 1 μg/mL biotinylated detection antibody for 1 hour. Plates were then washed and incubated with streptavidin–horseradish peroxidase (BioLegend, London, United Kingdom) for 20 minutes. After washing, TMB was used as a chromogenic substrate (Thermo Scientific). The reaction was stopped with 2N H_2SO_4, and OD was measured at 450 nm. A standard curve was obtained from a 7-point serial dilution of protein standard and used to calculate IL-36γ concentrations (Berekmeri et al., 2018). The lower limits of accurate detection for IL-8 and IL-36γ were 15.6 pg/ml and 24 pg/ml respectively.

FLAG Immunoprecipitation

Supernatants were centrifuged at 10000 g for removal of microbial and cellular debris and total protease inhibitor added to working concentration. M2-conjugated gel was washed and reconstituted to equivalent volume in PBS before adding 20 μl of M2-conjugated gel per ml of supernatants. Supernatants were then mixed for 24h at 4°C before pelleting of the M2-conjugated gel by gentle centrifugation. The pellet was washed in PBS, boiled in SDS-loading dye and subsequent analysis by western blot.

LC-MS

1 μg of protein was loaded onto a MassPREP micro desalting column (Waters) and washed for 5 min with 10% (vol/vol) acetonitrile/0.1% formic acid. Following a 1-min gradient to 85% (vol/vol) acetonitrile/0.1% formic acid, the protein was eluted into a Xevo G2-XS QToF (Waters) using electrospray ionization for molecular mass measurement.

Gel electrophoresis and western blotting

Samples were diluted in sample buffer (50 mM Tris HCl pH 6.8, 2% SDS, 10% glycerol, 0.02% bromophenol blue) and heated at 90°C for 5 min. Samples were resolved on a 15% (SUMO-tagged proteins) or 17% (immunoprecipitated samples) polyacrylamide gel and either stained with Coomassie or proteins transferred to a nitrocellulose membrane for western blotting. IL-36γ was detected using anti-IL-36γ antibody and avidin-HRP secondary. Proteins were visualized using enhanced chemiluminescence reagents (Sigma, UK).

QUANTIFICATION AND STATISTICAL ANALYSIS

Statistical analysis was performed using the software Graphpad Prism 7. Statistical details of experiments can be found in the figure legends. Data were analyzed by one-way ANOVA to determine overall differences and a Tukey post hoc test was performed to determine statistically significant differences between treatment groups. Differences were considered statistically significant when $p < 0.05$.

Cell Reports, Volume *33*

Supplemental Information

The Proinflammatory Cytokine IL-36γ Is a Global Discriminator of Harmless Microbes and Invasive Pathogens within Epithelial Tissues

Thomas Macleod, Joseph S. Ainscough, Christina Hesse, Sebastian Konzok, Armin Braun, Anna-Lena Buhl, Joerg Wenzel, Paul Bowyer, Yutaka Terao, Sarah Herrick, Miriam Wittmann, and Martin Stacey

Supplementary Information

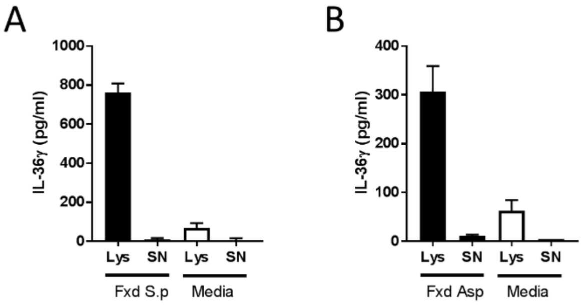

***Fig. S1**: TR146 cells do not release IL-36γ following non-viable pathogen stimulation,* related to Figure 1. TR146 cells (10^5 per well) were treated with fixed *S. pyogenes* (**A**; Fxd S.p), fixed *A. fumigatus* conidia **(B**; Fxd Asp) or media alone for 24 hours. Lysate and supernatant IL-36γ concentration measured by ELISA. Data shown are mean ± SEM (n = 3).

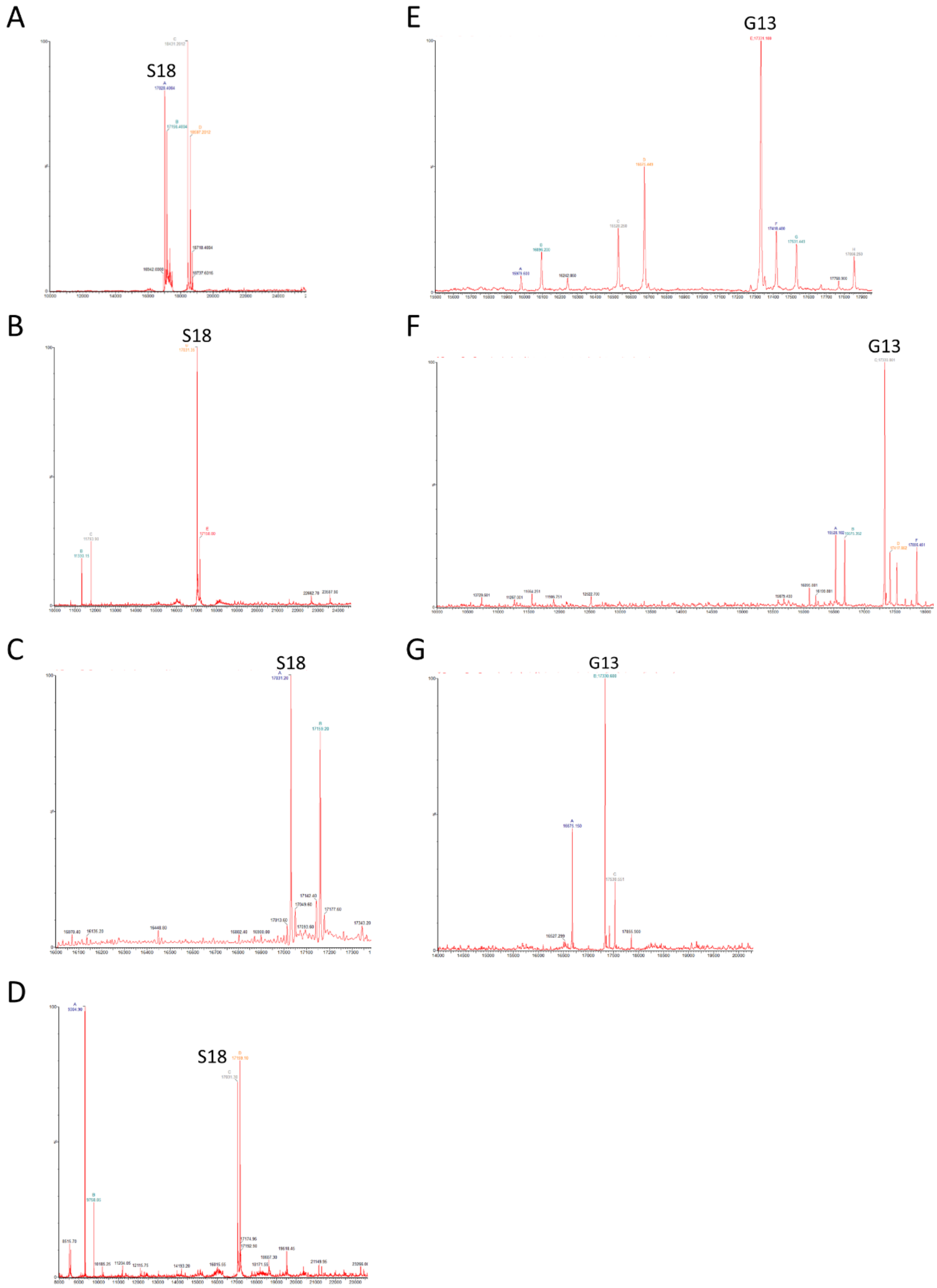

Fig. S2: *Liquid chromatography-mass spectrometry traces of IL-36 cleavage*, related to Figure 2. 2 µg of recombinant full length IL-36 proteins were incubated with culture filtrates or recombinant proteases for 1 hour at 37C and analysed by liquid chromatography-mass spectrometry as outlined in methods and materials. Liquid chromatography traces show resulting cleavage products from hIL-36γ + *A. fumigatus* (**A**), hIL-36γ + *T. rubrum* (**B**), hIL-36γ + *S. pyogenes* (**C**)**,** hIL-36γ + *S. areus* (**D**)**,** mIL-36γ + *A. fumigatus* (**E**), mIL-36γ + *T. rubrum* (**F**), mIL-36γ + *S. pyogenes* (**G**). Peaks corresponding to the active IL-36 truncations are depicted above and labelled with their respective N-terminal amino acids. hIL-36γ S18 (S18; 17031 Da), mIL-36γ G13 (G13; 17331 Da).

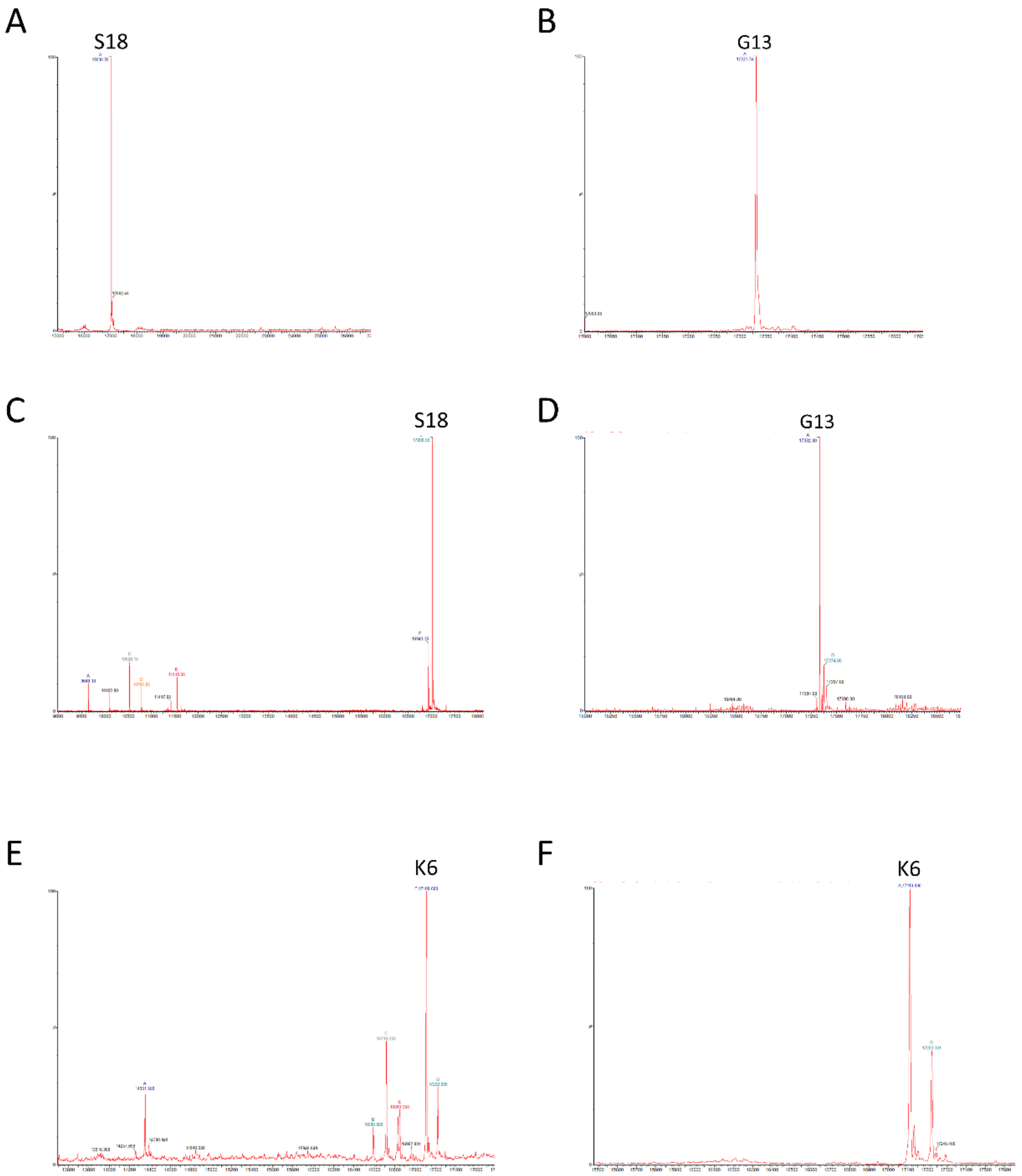

Fig. S3: *Liquid chromatography-mass spectrometry traces of IL-36 cleavage*, related to Figure 2. 2 µg of recombinant full length IL-36 proteins were incubated with culture filtrates or recombinant proteases for 1 hour at 37C and analysed by liquid chromatography-mass spectrometry as outlined in methods and materials. Liquid chromatography traces show resulting cleavage products from hIL-36γ + SpeB (**A**), mIL-36γ + SpeB (**B**), hIL-36γ + Asp F13 (**C**), mIL-36γ + Asp F13 (**D**), hIL-36α + *A. fumigatus* (**E**), hIL-36α + *T. rubrum* (**F**). Peaks corresponding to the active IL-36 truncations are depicted above and labelled with their respective N-terminal amino acids. hIL-36γ S18 (S18; 17031 Da), mIL-36γ G13 (G13; 17331 Da), hIL-36α K6 (K6; 17113).

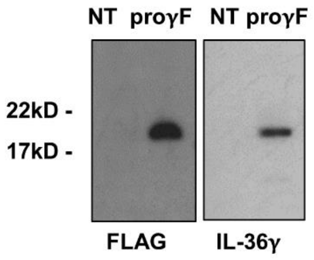

Fig. S4: *Confirmation of FLAG-tagged IL-36γ expression by stable proγF-293 cell line,* related to STAR Methods. Lysates of non-transfected 293 cells (NT) and stable proγF-293 cells (proγF) were analysed for expression of FLAG-tagged IL-36γ by western blot.

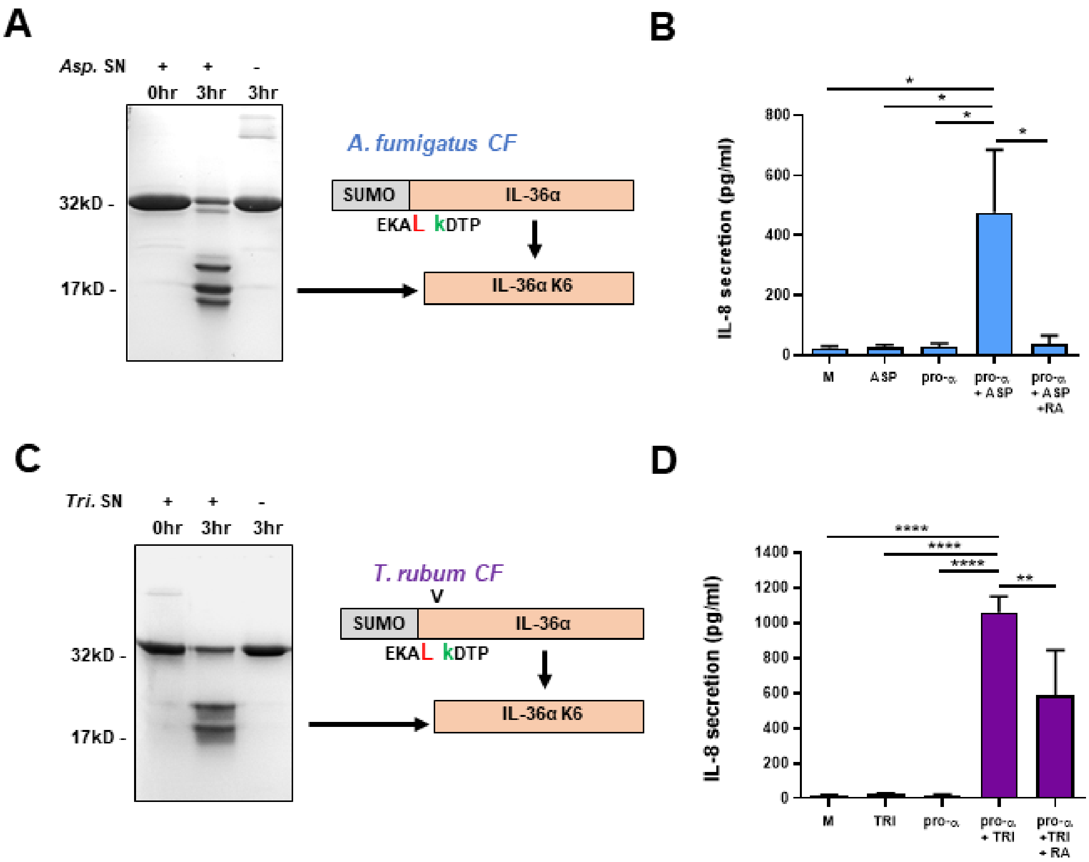

***Fig. S5**: IL-36a is also cleaved and activated by Aspergillus fumigatus and Trichophyton rubrum*, related to Figure 2. 1 µg of SUMO-tagged human IL-36α was incubated at 37˚C for 0 hours or 3 hours, with or without 2 µl of either *A. fumigatus* culture filtrate (Asp. CF; **A**) or *T. rubrum* culture filtrate (*Tri*. CF; **C**). Samples were analysed by Coomassie stained SDS-PAGE gel. Cleaved products were also analysed by mass spectrometry, with diagrams depicting the IL-36α truncation generated in response to each culture filtrate. In addition, HaCaT cells (10^5 per well) were incubated for 24 hours with media alone (M), pro-IL-36α (pro-α; 10 nM), culture filtrate, a combination of pro-IL-36α and culture filtrate, or a combination of pro-IL-36α, culture filtrate and IL-36RA (50 nM; **B, D**). A one-way ANOVA was used to determine statistical significance of differences between treatment groups. *$p < 0.05$, **$p<0.01$, ****$p < 0.0001$. Data shown are mean ± SEM (**B**; n=3, **D**; n=4).
